# Exergame-Based Behavior Change Interventions for Promoting Physical Activity: Systematic Review and Meta-Analysis of Randomized Controlled Studies

**DOI:** 10.2196/62906

**Published:** 2025-08-08

**Authors:** Si-Jia Li, Hao-Ming Ma, Lin-Qing Zhu, Hong-Yu Yu, Ao-Qi Wang, Xing-Yi Tang, Run-Yuan Pei, Mei-Hua Piao

**Affiliations:** 1 Chinese Academy of Medical Sciences, Peking Union Medical College School of Nursing, No. 33 Ba Da Chu Road, Shijingshan District, Beijing, 100144, China; 2 Department of Nursing, West China Hospital/West China School of Nursing, Sichuan University, Chengdu, China; 3 School of Nursing, University of Washington, Seattle, WA, United States

**Keywords:** exergame, physical activity, behavior change techniques, behavior change, systematic review, randomized controlled studies, promotion, noncommunicable diseases, life expectancy, exercise, cost-effective, video games, intervention

## Abstract

**Background:**

Physical inactivity is defined to be an activity level insufficient to meet recommendations. Exergame, which refers to a combination of exercise and video games, has the potential to promote physical activity (PA). Behavior change techniques (BCTs), the minimal, replicable components of an intervention, are widely used to identify components used in health behavior promotion.

**Objective:**

A systematic review and meta-analysis of randomized controlled trials (RCTs) was conducted to examine the overall effects of exergame-based interventions for promoting PA and their influencing factors. BCTs were also identified and discussed in this review.

**Methods:**

We searched for relevant RCTs across 6 databases from their inception to March 21, 2024. Meta-analyses using random-effects models assessed the effects on PA, moderate to vigorous physical activity (MVPA), light physical activity, moderate physical activity, vigorous physical activity, sedentary time, step count, and BMI. Subgroup analyses of PA were conducted to explore the influencing factors of exergame-based behavior change interventions. Review Manager software (version 5.3; Cochrane Collaboration) and Stata software (version 16; StataCorp) were used to analyze data.

**Results:**

A total of 20 RCTs targeting populations with various medical conditions (aged between 7.5 and 79 years; 1073/2211, 48.5% female) were included in this review, with sample sizes ranging from 16 to 1112. Exergame-based behavior change interventions significantly increased PA (standard mean difference [SMD] 0.19, 95% CI 0.05-0.33), MVPA (SMD 0.48, 95% CI 0.12-0.85), and step counts (SMD 0.54, 95% CI 0.13-0.94). Furthermore, subgroup analyses showed that intervention implementer (research assistants vs other implementers), game console (Microsoft Xbox vs Sony PlayStation vs Nintendo Wii), game participation type (individual game vs nonindividual game), measurement method (subjective vs objective), and the number of BCTs (n<7 vs 7≤n<10 vs n≥10) used significantly influenced the effectiveness of these interventions. The most frequently used BCTs included “1.4 action planning” (n=15), “1.1 goal setting” (n=13), “12.5 adding objects to the environment” (n=13), “2.3 self-monitoring of behavior” (n=11), and “4.1 instruction on how to perform the behavior” (n=11).

**Conclusions:**

Our review has demonstrated that exergame-based interventions are effective in promoting PA. Future trials are needed to further validate the insights proposed in our studies and assess the long-term effects on PA.

## Introduction

Insufficient physical activity (PA) represents a global public health problem, with significant health care costs and economic burdens [1]. Physical inactivity is defined to be an activity level insufficient to meet recommendations [2], which is estimated to be responsible for 6%‐10% of the major noncommunicable diseases (eg, coronary heart disease and type 2 diabetes) worldwide. Eliminating physical inactivity could potentially extend global life expectancy by 0.68 years [3]. It is conservatively estimated that physical inactivity cost global health care systems approximately US $53.8 billion in 2013 [4]. Considering the health impacts and financial costs of physical inactivity, promoting PA is essential.

With the advent and growing popularity of technology, digital health interventions (eg, mobile health apps, web-based health programs, and virtual reality fitness practices) have been increasingly used to promote PA, though their effectiveness varies due to differing delivery methods [5,6]. According to the results of qualitative interviews and cross-sectional surveys, lack of motivation is recognized as a major barrier to promoting PA [7]. Exergame, a form of digital health intervention that combines exercise with video games, has the potential to overcome this barrier, as people are more likely to experience a state of flow while participating in exergame activities [8-10]. Games, such as Dance Dance Revolution, invented by the Japanese company Konami, and interactive cycling, offer a convenient, cost-effective, and engaging alternative to traditional exercise, potentially increasing motivation for PA [11]. In recent years, exergames have attracted researchers’ attention as they examine the health benefits of exergames, which are initially designed for entertainment. Many studies examining their effectiveness have been conducted. For instance, in 2019, a randomized controlled trial (RCT) investigated the effect of Nintendo Wii Fit on promoting PA in children with cancer, but no significant PA benefits were observed [12]. Another RCT, involving a 5-month exergame training intervention for children with overweight or obesity, revealed significant increases in PA and a decrease in sedentary time (SED) [13]. The inconsistency in the original research findings has led researchers to question the true effects of exergames and prompted them to conduct meta-analyses.

A systematic review and meta-analysis regarding the effectiveness of exergame interventions targeting PA behaviors was published in 2023 [8]. However, there are some limitations to this paper. First, the inclusion of both RCTs and quasi-experimental studies may compromise the reliability of the evidence presented. Second, only articles published before December 31, 2020, are included. Yet, numerous original studies on the effectiveness of exergame-based interventions for promoting PA have emerged from 2021 to 2024, indicating that the evidence needs to be updated [13-18]. Third, effective exergame-based intervention components are not fully explored and discussed in the previous review. Another review relevant to this topic was also published recently, but it also did not fully address the components of exergame interventions. It primarily focused on the intervention period, population, outcome measurement, and control group type, which are important but are commonly discussed in most behavior change articles [19]. Understanding the characteristics that distinguish exergames from other behavior change interventions (eg, primary game device, console type, participation type, and content) is essential, as they are likely to be underlying psychological factors that drive the effectiveness of exergames. Clarifying the effective components used in exergame-based intervention design also helps guide the development of future interventions aimed at improving health outcomes. To make the use of intervention components clearer and more explicit, we introduced behavior change techniques (BCTs) in this systematic review [20]. According to Michie et al [21], BCTs are systematically categorized into 93 techniques, such as goal setting, self-monitoring, and feedback. They are the minimal, replicable components of an intervention designed to influence or modify the causal processes that regulate behavior and are widely used to identify components involved in health behavior promotion [21-24].

Therefore, we aimed to investigate the overall effectiveness of exergame-based behavior change interventions on PA promotion and explore potential factors, especially exergame-specific ones, that influence their effectiveness, with the goal of guiding future intervention design. Specifically, this systematic review and meta-analysis of RCTs was performed to examine the effects of exergame-based interventions on primary outcomes (ie, PA) and secondary outcomes (ie, moderate to vigorous physical activity [MVPA], moderate physical activity [MPA], vigorous physical activity [VPA], light physical activity [LPA], SED, step count, and BMI). Furthermore, we conducted subgroup analyses to identify factors that significantly influence the effectiveness of exergame-based interventions. BCTs were also identified and discussed in this review.

## Methods

### Overview

The systematic review and meta-analysis were conducted following the PRISMA (Preferred Reporting Items for Systematic Reviews and Meta-Analyses) 2020 guidelines [25] and were registered on the International Prospective Register of Systematic Reviews (PROSPERO) with registration number CRD42024544081.

### Search Strategy

We searched 6 databases (ie, PubMed, Embase, Cochrane Library, Web of Science, CINAHL, and SPORTDiscus) in English for relevant RCTs published from the inception of each database to March 21, 2024. The search strategy was developed with the assistance of a research librarian based on the PICOS (Participants, Interventions, Comparisons, Outcomes, and Study Design) framework. MeSH (Medical Subject Headings) and key search terms related to “exergaming,” “exergame*,” “active-video gam*,” “exercise,” “physical activit*,” and “randomized controlled trial” were included. Additionally, to ensure comprehensive retrieval, we manually searched the references of previously published relevant reviews (Multimedia Appendix 1).

### Study Eligibility Criteria

The inclusion criteria for this systematic review and meta-analysis were as follows: (1) participants: any human population, irrespective of health condition; (2) interventions: exergame used either as the primary intervention or as a supplementary component; (3) comparisons: either active controls (eg, PA education programs) or inactive controls (eg, no intervention and waitlist control); (4) outcomes: PA outcomes measured both objectively, including minutes of various types of PA, and subjectively through assessment tools such as the International Physical Activity Questionnaire; in addition, BMI was also included as an indirect measure of PA effects; and (5) studies: RCTs of all forms, including parallel, cluster, and crossover designs. Conference papers and abstracts were excluded to ensure data quality, and full texts lacking available data were also excluded.

### Study Selection

All studies retrieved were imported into EndNote 20 (Clarivate), and duplicates were automatically removed. The results were then exported to the Rayyan web-based platform [26]. Two authors (SJL and LQZ) independently screened the titles and abstracts based on the eligibility criteria. Next, full texts of potentially relevant articles were retrieved and assessed for inclusion, with reasons for exclusion recorded. Any discrepancies were resolved through discussions with a third investigator (HMM) to reach a consensus.

### Data Extraction

Study characteristics (including author, year, country, and study arms), population details (including population type, sample size, average years, sex difference, and baseline BMI), intervention specifics (including theory, site, frequency, type of training, duration, and period), exergame characteristics (including game name, primary device, and content), brief comparison content, and outcome data were extracted into a predesigned Microsoft Excel format by one author (SJL) and cross-checked by another reviewer (LQZ). The mean plus SD format was used to represent outcome data, and other statistics were converted to mean (SD) format [27]. Median values were transformed into means if mean values were not reported in the original articles [28]. Additionally, other data formats (such as 95% CI or standard errors) were also converted into SD (*Cochrane Handbook*, version 6.4, Chapter 5) [27]. If outcome indicators had different units, such as using metabolic equivalent of task to measure PA, we applied conversion formulas mentioned in the methods section of the articles to convert them into minutes per day, ensuring consistency in units. If a study involved multiple study arms, each individual intervention was treated as a separate study, and the number of participants in the shared control group was divided by the number of study arms to avoid duplicate inclusion of participants in the meta-analysis (*Cochrane Handbook*, version 6.4, Chapter 23) [27]. Michie’s BCT taxonomy (BCTTv1) was used to identify BCTs used in the original studies (Multimedia Appendix 2) [21]. If intervention descriptors in original RCTs matched the definitions of BCTs, we assigned a “√.” One reviewer (SJL) initially identified the BCTs, which were then double-checked and confirmed by another reviewer (HMM).

### Risk of Bias Assessment

Two independent reviewers (SJL and LQZ) evaluated the methodological quality of the included RCTs using version 1 of the Cochrane risk of bias tool [29]. This tool consisted of 7 categories: random sequence generation (selection bias), allocation concealment (selection bias), blinding of participants and personnel (performance bias), blinding of outcome assessment (detection bias), incomplete outcome data (attrition bias), selective reporting (reporting bias), and other potential biases such as conflicts of interest. Each domain was assessed and rated as having low, unclear, or high risk of bias. Any discrepancies during the evaluation process were resolved by a third investigator (HMM).

### Statistical Analysis

The meta-analysis aimed to assess the effectiveness of exergame-based behavior change interventions on promoting PA. Total PA, defined as the sum of LPA, MPA, and VPA measured either by a scale or an accelerometer, was the primary outcome, with MVPA, LPA, MPA, VPA, SED, step count, and BMI as secondary outcomes. Given the diverse measurement methods for outcomes, we calculated standard mean differences (SMDs), estimated using adjusted Hedges *g*, to generate effect sizes [30,31]. SMD values of 0.2, 0.5, and 0.8 corresponded to small, medium, and large effects, respectively [32]. Data heterogeneity was evaluated using the *I*² statistic, categorizing values of 25%, 50%, or 75% as low, moderate, and high, respectively [33]. A random-effects model was used for data analysis to obtain conservative results. Publication bias was assessed through visual evaluation of funnel plots asymmetry and quantified using Egger tests. Publication bias assessments were conducted only for outcome measures that included more than 10 original studies (*Cochrane Handbook*, version 6.4, Chapter 13) [27]. Subgroup analyses were conducted to explore influencing factors of exergame-based behavior change interventions and potential sources of heterogeneity. Review Manager software (version 5.3; Cochrane Collaboration) and Stata software (version 16; StataCorp) were used to analyze data. In all analyses, *P*<.05 (2-sided) was considered statistically significant.

## Results

### Study Selection

**Figure 1.**
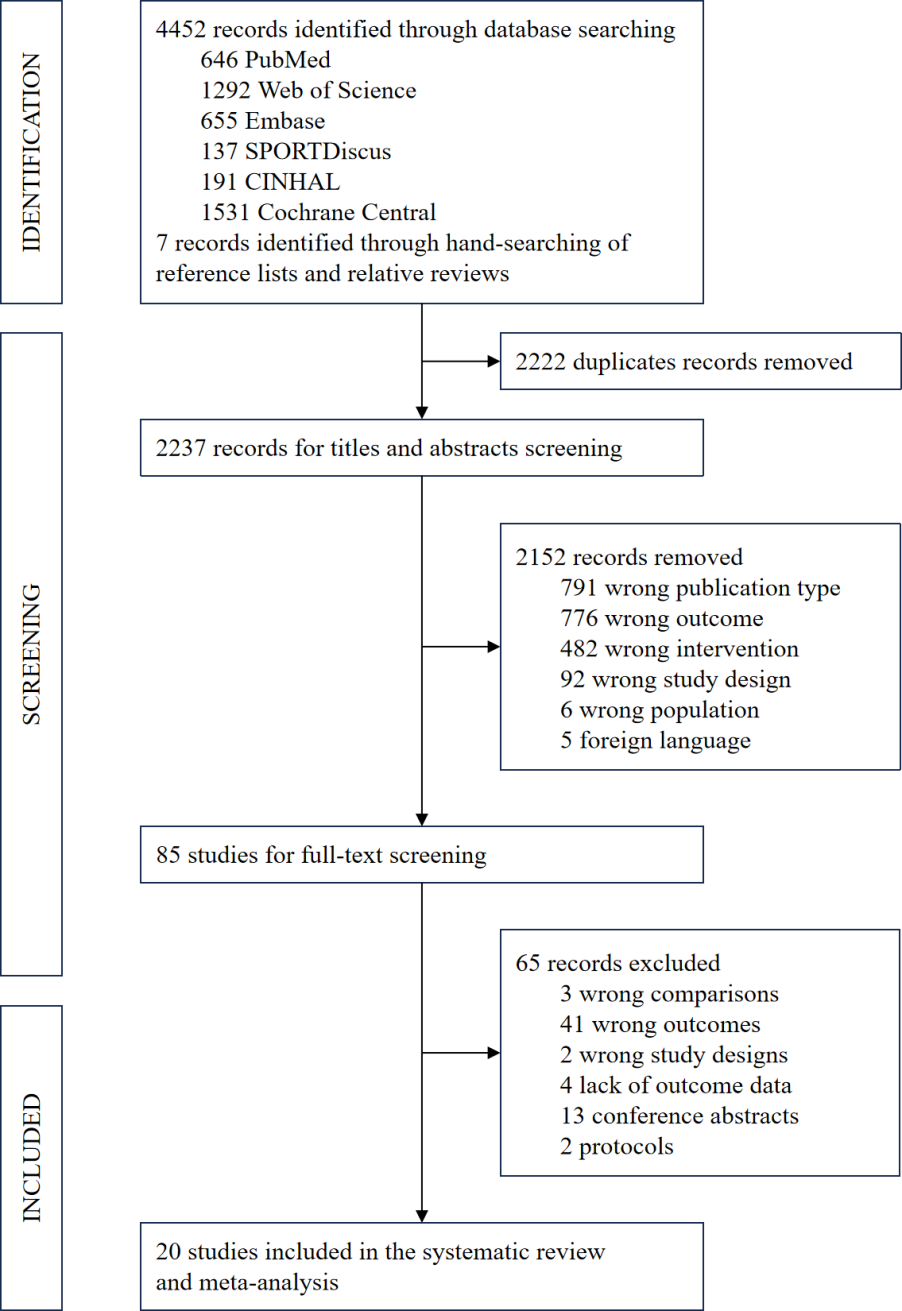
PRISMA (Preferred Reporting Items for Systematic Reviews and Meta-Analyses) flow diagram of the study selection process.

The literature search initially yielded 4452 records. Additionally, 7 studies were identified by hand-searching the references of relevant reviews. After the removal of 2222 duplicates using EndNote 20, titles and abstracts of 2237 studies were screened. Subsequently, 85 studies were included for full text screening, with 65 being excluded due to wrong interventions (n=1), wrong comparisons (n=3), wrong outcomes (n=41), wrong study designs (n=2), lack of outcome data (n=4), conference abstracts (n=13), and 2 protocols (see Multimedia Appendix 3 for a detailed list of exclusions with corresponding reasons). Finally, 20 RCTs [12-18,34-46] were included in the systematic review and meta-analysis (see Figure 1).

### Study Characteristics

The studies included in the review were published between 2008 and 2023. A total of 20 studies were performed in Canada [15,34,37], the United States [14,17,18,35,36,42,43], Turkey [16,44], Spain [13], Australia [38,45], China (Hong Kong) [40], the Netherlands [46], Germany [39], Singapore [41], and Finland [12]. Two studies [15,17] used a 3-arm RCT design, while the remaining studies [12-14,16,18,34-46] used a 2-arm RCT design. Among them, 5 studies [15,18,36,44,46] focused on adults and 15 studies [12-14,16,17,34,35,37-43,45] targeted children. The sample sizes varied from 16 to 1112, with participants’ mean ages ranging from 7.5 to 79 years. The review included 2211 participants for meta-analysis, with 48.5% (n=1073) being female. BMI was also recorded to provide a general assessment of the participants’ body composition. Detailed characteristics of the studies and populations are listed in Table 1.

**Table 1.** Characteristics of studies and populations.

Reference	Country	Study arms	Population (IG/CG)^a^^,^^b^				
			Population type	Sample size (n)	Age (years), mean (SD)	Sex difference (male/female)	BMI (kg/m^2^) , mean (SD)
Adamo et al [34]	Canada	2-arm RCT^c^; IG: interactive video game cycling; CG: stationary cycling to music	Overweight and obese adolescents	26 (13/13)	15.1 (1.8)/13.9 (1.4)	12/14	39.3 (9.0)/35.5 (9.3)
Baranowski et al [35]	United States	2-arm RCT; IG: active video games; CG: inactive video games	Children	84 (41/43)	11.3 (1.8)	43/41	BMI percentile: 81.7%
Campelo et al [15]	Canada	3-arm RCT; IG: exergame training; CG1: conventional training; CG2: no training	Older adults	IG/CG1/CG2: 40 (15/14/11)	IG/CG1/CG2: 72.6 (7.7)/71.9 (5.0)/73.5 (7.5)	15/25	IG/CG1/CG2: 30.6 (5.7)/28.0 (4.7)/25.6 (3.5)
Cavusoglu et al [16]	Turkey	2-arm RCT; IG: Nintendo Wii Fit; CG: home-based fun video exercises	Pediatric patients with chronic kidney disease	16 (8/8)	11.5 (3.5)/11.1 (3.7)	5/11	18.4 (5.5)/18.0 (4.6)
Comeras-Chueca et al [13]	Spain	2-arm RCT; IG: active video games exercise program combined with multicomponent exercise; CG: continue daily activities	Children with overweight or obesity	29 (21/8)	10.2 (0.8)/9.7 (0.8)	16/13	25.9 (2.9)/24.1 (1.7)
Garde et al [37]	Canada	2-arm crossover RCT; IG: mobile exergame (MobileKids Monster Manor); CG: no intervention	Healthy students	42 (42/42)	11.3 (1.2)/11.5 (1.3)	26/16	BMI z-score: 0.6 (1.4)/0.1 (1.2)
Howie et al [38]	Australia	2-arm RCT; IG: active video games; CG: no active video games	Children with DCD^d^ or at risk for DCD	21 (10/11)	11.0 (1.0)	10/11	BMI percentile: 75% (24%)
Lau et al [40]	China (Hong Kong)	2-arm RCT; IG: active video games; CG: no intervention	Children	80 (40/40)	9.2 (0.5)	55/25	19.4 (3.6)/19.8 (3.6)
Sousa et al [17]	United States	3-arm crossover RCT; IG: active VR^e^; CG 1: sedentary VR; CG 2: control group	College students	IG/CG1/CG2: 29 (29/29/29)	23.2 (2.1)	20/9	23.7 (4.0)
Swartz et al [18]	United States	2-arm RCT; IG: active video game sessions; CG: standard care	Female survivors of breast cancer	60 (30/30)	56.1 (10.7)/58.7 (10.3)	0/60	29.4 (6.2)/31.8 (8.5)
Trost et al [45]	Australia	2-arm RCT; IG: program and active gaming intervention; CG: program-only intervention	Children with a BMI >85th percentile	75 (34/41)	10.1 (1.9)/9.9 (1.5)	34/41	28.4 (6.7)/27.3 (3.6)
van Santen et al [46]	The Netherlands	2-arm RCT; IG: exergame intervention; CG: care-as-usual control group	People with dementia	112 (73/39)	79.0 (6.0)/79.0 (7.0)	60/52	28.0 (4.7)/29.0 (5.5)
Maloney et al [42]	United States	2-arm RCT; IG: DDR^f^; CG: waitlist control	Children	60 (40/20)	7.5 (0.5)/7.6 (0.5)	30/30	17.2 (2.4)/18.0 (3.3)
Maloney et al [43]	United States	2-arm RCT; IG: DDR+ pedometers; CG: pedometers only	Children with over 85th percentile for BMI	64 (33/31)	12.9 (2.4)/11.7 (2.4)	30/34	BMI percentile: 96.6% (3.7%)/96.6% (2.7%)
Şimşek and Çekok [44]	Turkey	2-arm RCT; IG: Nintendo Wii group; CG: Bobath neurodevelopmental treatment	Patients with subacute stroke	42 (20/22)	54.2 (20.3)/61.5 (11.6)	29/13	24.4 (3.7)/28.2 (5.2)
Bowling et al [14]	United States	2-arm RCT; IG: AGS^g^ intervention; CG: waitlist control	Youths with neurodevelopmental and psychiatric diagnosis	23 (11/12)	15.3/14.9	17/6	—^h^
Kempf and Martin [39]	Germany	2-arm RCT; IG: exercise game Wii Fit Plus; CG: routine care	Type 2 diabetes patients	220 (120/100)	62.0 (11.0)/60.0 (9.0)	101/119	34.1 (6.5)/33.2 (6.3)
Lwin and Malik [41]	Singapore	2-arm RCT; IG: PE^i^ lesson with Wii; CG: PE lesson without Wii	Children and preadolescents	1112 (557/555)	11.3 (1.1)	603/509	—
Cowdery et al [36]	United States	2-arm RCT; IG: exergame smartphone apps; CG: activity tracking app	People aged 18‐69 years	40 (20/20)	31.8 (4.9)/33.0 (5.5)	6/34	Weight: 167.0 (11.1)/171.4 (22.1)
Hamari et al [12]	Finland	2-arm RCT; IG: Nintendo Wii Fit; CG: PA^j^ advice	Children with cancer	36 (17/19)	7.8 (3.3)/7.9 (3.0)	26/10	—

a IG: intervention group.

b CG: control group.

c RCT: randomized controlled trial.

d DCD: developmental coordination disorder.

e VR: virtual reality.

f DDR: Dance Dance Revolution.

g AGS: adaptive GameSquad.

h Not applicable.

i PE: physical education.

j PA: physical activity.

### Characteristics of Exergame-Based Interventions

The design of some exergame-based interventions incorporated theories (ie, self-determination theory, social cognitive theory, and the theory of planned behavior) or theoretical frameworks (reserve capacity model and family ecological model). Interventions took place either at home or outside the home, such as in a hospital. The type of training ranged from simple activities such as walking and cycling to complex multicomponent exercises. The intervention period spanned from 1 to 24 weeks, with frequencies ranging from 1 to 7 times per week, each lasting between 20 and 60 minutes. Measured outcomes included PA, MVPA, LPA, MPA, VPA, SED, step count, and BMI, as detailed in Table 2. Regarding the characteristics of exergames, a total of 50 exergames (eg, Game Bike, Dance Dance Revolution, and Wii Sports) were identified across the 20 RCTs. Sony PlayStation, Nintendo Wii, and Microsoft Xbox were the primary devices used in these exergames. The game content included a diverse range of activities such as cycling, sports, dance, aerobics, strength training, yoga, adventure, walking, mini-games, pet simulation, and rhythm-based games. Detailed information about the exergames can be found in Multimedia Appendix 4 [12-18,34-46].

**Table 2.** Characteristics of interventions and outcomes.

Reference	Intervention						Outcome
	Theory	Site	Frequency (time/week)	Type of training	Duration (minutes)	Period (weeks)	
Adamo et al [34]	—^a^	Lab	2x/week	Cycling	60	10	SED^b^, LPA^c^, MVPA^d^, step count
Baranowski et al [35]	Self-determination theory	Home	—	—	—	12	Step count
Campelo et al [15]	—	Living center	3x/week	Aerobics, strength, balance, and flexibility	40	6	Step count
Cavusoglu et al [16]	—	Clinical setting	2x/week	Aerobics, strengthening, and yoga exercises	40	6	SED, LPA, MPA^e^, VPA^f^, MVPA, PA^g^, BMI
Comeras-Chueca et al [13]	—	University and public school	3x/week	Multicomponent exercises	60	20	Step count
Garde et al [37]	—	Inside and outside the school environment	—	—	—	1	PA, SED, LPA, MPA, VPA
Howie et al [38]	—	Home	4‐5x/week	Targeted a variety of gross and fine motor skills	20	16	PA, MVPA, BMI
Lau et al [40]	—	School	2x/week	Moderate intensity activity	60	12	MVPA
Sousa et al [17]	—	Lab	—	Upper-body movement or whole-body movement	20	—	Step count, LPA, MVPA
Swartz et al [18]	Social cognitive theory, self-determination theory	Clinical setting	3x/week	Whole-body movement	60	12	MVPA, VPA, BMI z score
Trost et al [45]	—	YMCA^h^ + school	1x/week	Whole-body movement	60	16	PA
van Santen et al [46]	—	Day care centers	2x/week	Interactive cycling	—	24	PA, SED, LPA, MPA, VPA, BMI
Maloney et al [42]	—	Home	4x/week	Dance	30	10	Step count, LPA, MPA, VPA
Maloney et al [43]	—	Home	—	Dance	—	12	PA
Şimşek and Çekok [44]	—	Hospital	3x/week	Upper limb and balance training	45‐60	10	PA
Bowling et al [14]	Reserve capacity model and family ecological model	—	3x/week	Whole-body movement	—	10	BMI
Kempf and Martin [39]	—	—	7x/week	Strength and yoga activities	30	12	PA, LPA, MPA, VPA
Lwin and Malik [41]	Theory of planned behavior	School	1x/week	Whole-body movement	45‐60	6	PA, MPA, VPA, LPA, BMI
Cowdery et al [36]	Self-determination theory	—	—	Walk and run	—	12	Step count, PA
Hamari et al [12]	—	Hospital and home	7x/week	—	30	8	BMI

a Not applicable.

b SED: sedentary time.

c LPA: light physical activity.

d MVPA: moderate to vigorous physical activity.

e MPA: moderate physical activity.

f VPA: vigorous physical activity.

g PA: physical activity.

h YMCA: Young Men's Christian Association.

### The Frequency of BCT Used in These Interventions

The application of BCTs in the included RCTs varied, with each study incorporating between 1 and 15 different techniques. The most frequently used BCTs, which appeared in more than half of the studies, included “1.4 action planning” (n=15), “1.1 goal setting” (n=13), “12.5 adding objects to the environment” (n=13), “2.3 self-monitoring of behavior” (n=11), and “4.1 instruction on how to perform the behavior” (n=11) (shown in Table 3 and Multimedia Appendix 5).

**Table 3.** Frequency of BCTs^a^.

BCT taxonomy	Studies used the BCT, n (%)
1.4 Action planning	15 (75)
1.1 Goal setting (behavior)	13 (65)
12.5 Adding objects to the environment	13 (65)
2.3 Self-monitoring of behavior	11 (55)
4.1 Instruction on how to perform the behavior	11 (55)
2.2 Feedback on behavior	9 (45)
3.1 Social support (unspecified)	8 (40)
8.7 Graded tasks	8 (40)
1.2 Problem-solving	6 (30)
3.2 Social support (practical)	6 (30)
2.1 Monitoring of behavior by others without feedback	4 (20)
6.1 Demonstration of the behavior	4 (20)
6.2 Social comparison	3 (15)
9.1 Credible source	3 (15)
10.3 Nonspecific reward	3 (15)
11.3 Conserving mental resources	3 (15)
12.1 Restructuring the physical environment	3 (15)
10.2 Material reward (behavior)	2 (10)
10.4 Social reward	2 (10)
1.5 Review behavior goals	1 (5)
2.5 Monitoring of outcomes of behavior without feedback	1 (5)
3.3 Social support (emotional)	1 (5)
5.1 Information about health consequences	1 (5)
5.6 Information about emotional consequences	1 (5)
7.1 Prompts/cues	1 (5)
8.1 Behavioral practice/rehearsal	1 (5)
12.2 Restructuring the social environment	1 (5)
14.4 Reward approximation	1 (5)
14.5 Rewarding completion	1 (5)

a BCTs: behavior change techniques.

### Risk of Bias Assessment

**Figure 2.**
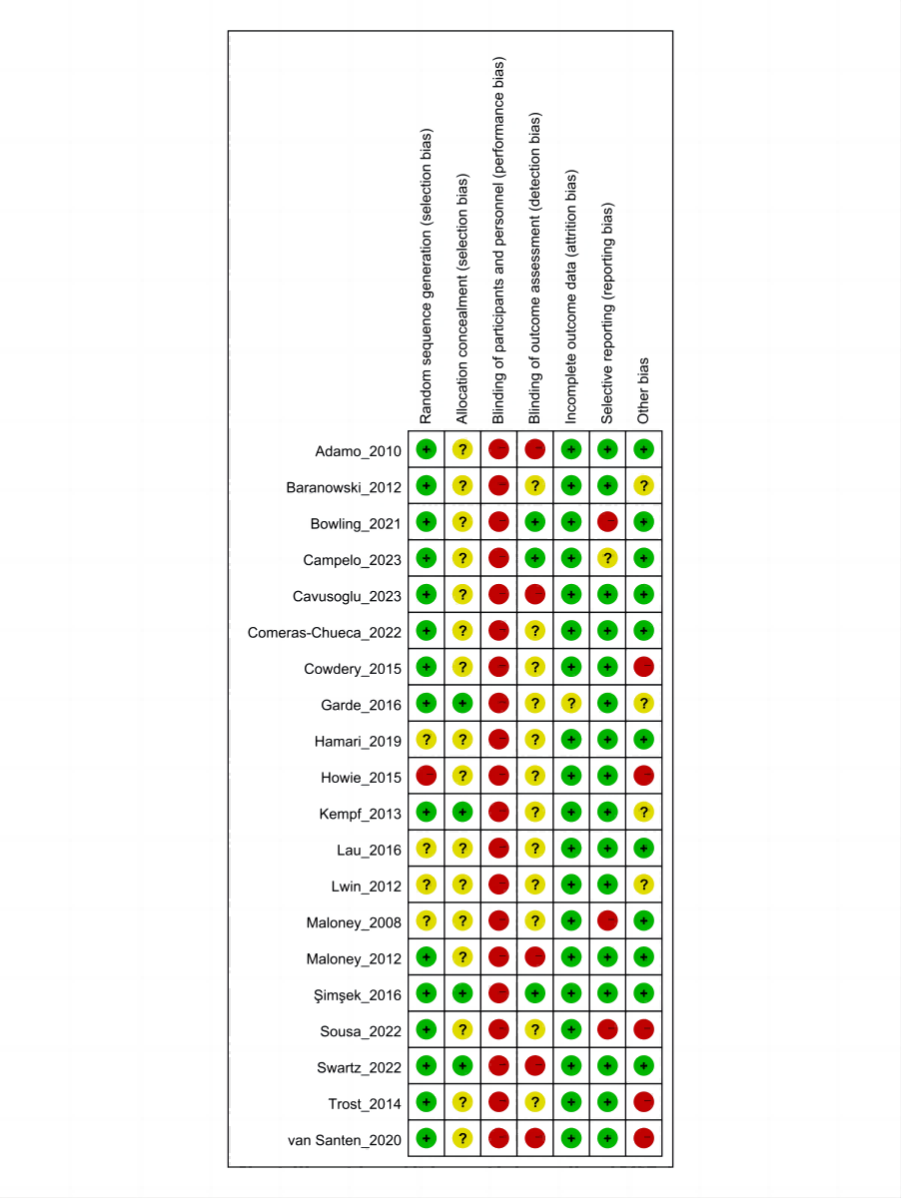
Risk of bias [12-18,34-46].

A vast majority (15/20, 75%) of the studies explicitly described the generation of random sequences, while those that mentioned randomization without detailing the process were categorized as unclear. Only 4 studies provided the specific details on the methods of allocation concealment, such as using opaque envelopes to conceal the random sequences. Due to the nature of behavior change intervention studies, it is unlikely to blind participants. Therefore, all studies were rated as high risk in this category. Information regarding the blinding of outcome assessment was missing in 60% (12/20) of the studies. Only one study was assessed as unclear in the incomplete outcome data section due to an insufficient explanation for the missing data. Most studies were categorized as low risk in the selective reporting and other potential biases. Detailed risk of bias assessment results can be seen in Figure 2.

### Effectiveness of Exergame-Based Interventions

The systematic review and meta-analysis assessed the effectiveness of exergame-based interventions on primary outcomes, specifically PA, as well as on secondary outcomes, including MVPA, LPA, MPA, VPA, SED, step count, and BMI. Additionally, subgroup analyses were performed to identify potential factors influencing the effectiveness of the interventions.

### Meta-Analysis of Primary Outcomes (PA)

**Figure 3.**
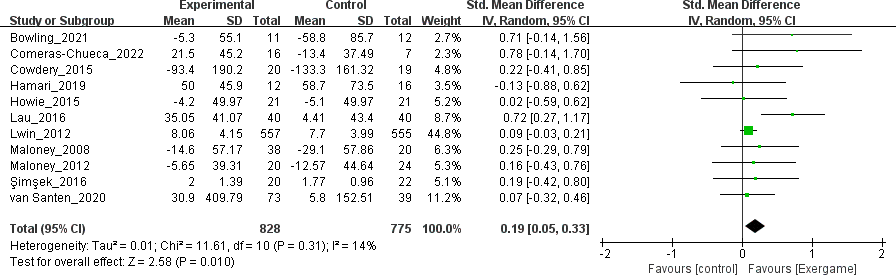
Forest plot of physical activity [12-14,36,38,40-44,46].

**Figure 4.**
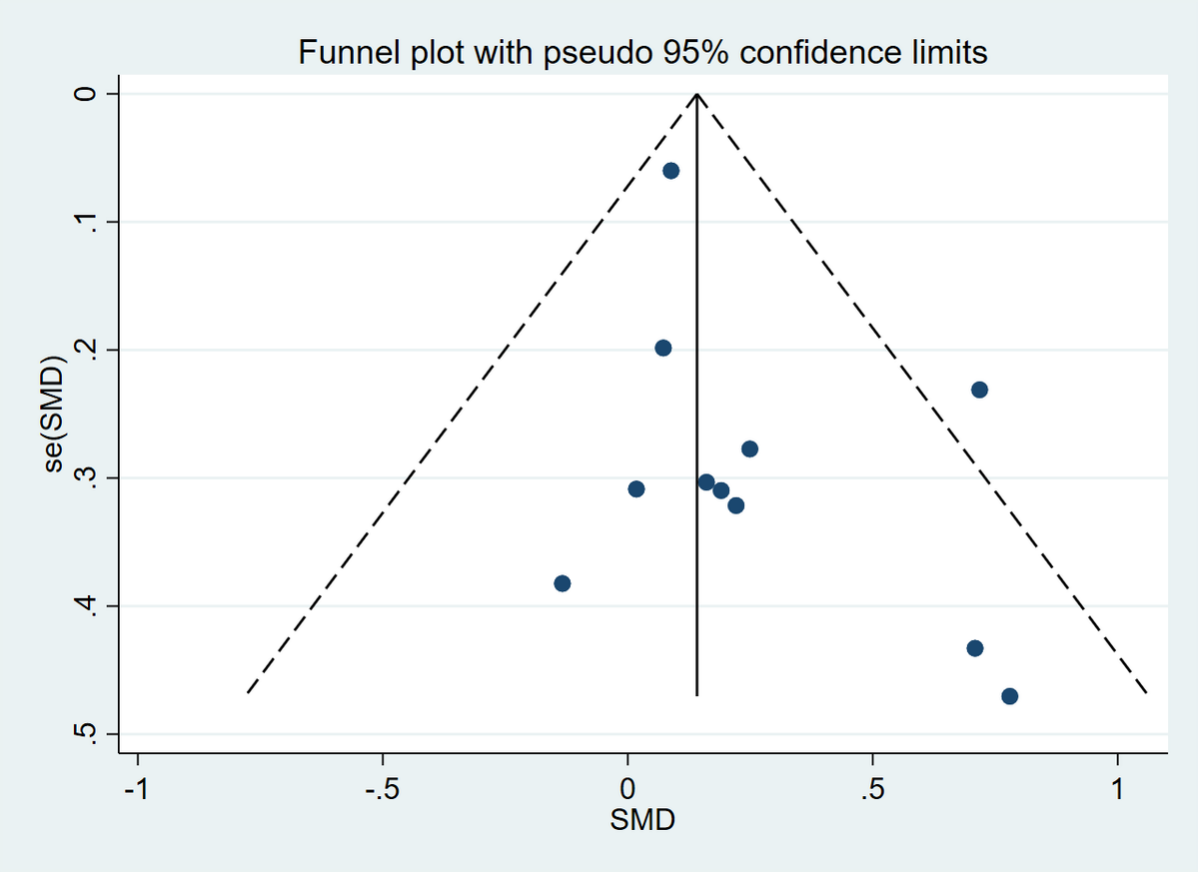
Funnel plot of physical activity. se: standard error; SMD: standard mean difference.

A total of 11 studies [12-14,36,38,40-44,46], including 828 participants in the intervention groups and 775 participants in the control groups, were analyzed to investigate the effects of exergame-based interventions on PA. The pooled effect size of PA was 0.19 (95% CI 0.05-0.33), indicating a statistically significant small effect. Additionally, a low level of heterogeneity was observed (*I*^2^=14%) (see Figure 3). No publication bias was detected by the Egger test (*P*=.12). The funnel plot is displayed in Figure 4.

### Subgroup Analysis and Meta-Regression of Primary Outcomes (PA)

Sixteen subgroup analyses were conducted to evaluate the effects of exergame-based interventions: population (age group [children vs adults] and health status [healthy participants vs participants with health conditions]), intervention design (theoretical basis [theory-based vs nontheory-based], site [school vs home vs hospital], frequency [4x/week vs ≥4x/week], session duration [<40 minutes vs ≥40 minutes], intervention period [<3 months vs ≥3 months], family involvement [yes vs no], technology support [entirely vs partially]), and intervention implementer [research assistants vs other implementers]), exergame design (game primary device [console vs smartphone vs cycling simulator], game console [Xbox, PlayStation, and Nintendo Wii], game participation type [individual game vs nonindividual game], and game content [single-category vs multicategory]), PA measurement (subjective vs objective), and BCT number used (n<7 vs 7≤n<10 vs n≥10).

Intervention implementer, game console, game participation type, measurement methods, and the number of BCTs used in each study significantly influenced the effects of exergame-based interventions on PA. Compared to other study implementers (eg, physical therapists), studies implemented by research assistants exhibited statistically significant higher effect sizes (SMD 0.36 vs 0.09, *P*=.04); similarly, the Xbox group and the nonindividual game group achieved significantly higher effect sizes than the PlayStation and Nintendo Wii group (SMD 0.72 vs 0.21 vs 0.09, *P*=.01), and the individual game group (SMD 0.46 vs 0.10, *P*=.02), respectively. Additionally, objectively measured PA demonstrated a larger effect size (SMD 0.41) compared to subjectively measured PA (SMD 0.09, *P*=.03). Similarly, studies using 10 or more BCTs showed a greater effect size (SMD 0.55) than those in the other groups.

Other important variables, such as age groups, intervention period, and game content, warrant our attention. Although they did not reveal significant subgroup differences, children showed significantly greater improvements in PA compared to adults (SMD 0.26, 95% CI 0.03 to 0.49 vs SMD 0.13, 95% CI −0.16 to 0.42). Interventions lasting longer than 3 months demonstrated significantly better PA outcomes than those shorter than 3 months (SMD 0.29, 95% CI 0.03 to 0.56 vs SMD 0.10, 95% CI −0.01 to 0.21). Single-category game content had significantly better PA outcomes than multicategory game content, such as combining dance and sports (SMD 0.29, 95% CI 0.04 to 0.54 vs SMD 0.13, 95% CI −0.02 to 0.28). Detailed subgroup analyses are listed in Table 4.

**Table 4.** Subgroup analysis of physical activity.

Variables and subgroup	Studies, n	SMD^a^ (95% CI)	*I*^2^ (%)	*P* value of subgroup difference
Population				
Age group				.50
Children	8	0.26 (0.03 to 0.49)	39	
Adults	3	0.13 (–0.16 to 0.42)	0	
Health status				.54
Healthy participants	4	0.28 (–0.03 to 0.60)	58	
Participants with health conditions	7	0.16 (–0.06 to 0.39)	0	
Intervention design				
Theory				.36
Theory-based	3	0.12 (–0.05 to 0.30)	7	
Nontheory-based	8	0.25 (0.04 to 0.46)	11	
Site				.50
School	3	0.44 (–0.09 to 0.97)	77	
Home	3	0.11 (–0.17 to 0.39)	0	
Hospital	2	0.06 (–0.41 to 0.53)	0	
Frequency				.33
<4x/week	6	0.31 (0.04 to 0.57)	53	
≥4x/week	3	0.08 (–0.27 to 0.44)	0	
Duration				.30
<40 min	3	0.08 (–0.27 to 0.44)	0	
≥40 min	4	0.36 (–0.03 to 0.75)	66	
Period				.19
<3 months	5	0.10 (–0.01 to 0.21)	0	
≥3 months	6	0.29 (0.03 to 0.56)	26	
Family involvement				.99
Yes	4	0.22 (–0.11 to 0.54)	0	
No	7	0.22 (0.01 to 0.42)	35	
Technology support				.53
Entirely	6	0.25 (0.03 to 0.47)	11	
Partially	5	0.15 (–0.05 to 0.36)	13	
Intervention implementer				.04
Research assistants	6	0.36 (0.12 to 0.60)	1	
Other implementers	4	0.09 (–0.02 to 0.20)	0	
Exergame design				
Game primary device				.75
Console	8	0.24 (0.04 to 0.44)	34	
Smartphone	1	0.22 (–0.41 to 0.85)	—^b^	
Cycling simulator	1	0.07 (–0.32 to 0.46)	—	
Game console				.01
Xbox	2	0.72 (0.32 to 1.12)	0	
PlayStation	2	0.21 (–0.19 to 0.61)	0	
Nintendo Wii	3	0.09 (–0.03 to 0.20)	0	
Game participation type				.02
Individual game	7	0.10 (–0.01 to 0.20)	0	
Nonindividual game	4	0.46 (0.17 to 0.75)	4	
Game content				.28
Single-category	5	0.29 (0.04 to 0.54)	18	
Multicategory	5	0.13 (–0.02 to 0.28)	5	
Outcome measurement				
Measurement				.03
Subjective	4	0.09 (–0.02 to 0.20)	0	
Objective	6	0.41 (0.15 to 0.67)	9	
BCT^c^ used				
BCT number				.02
Low BCT use (n<7)	5	0.10 (–0.01 to 0.21)	0	
Moderate BCT use (7≤n<10)	3	0.00 (–0.29 to 0.29)	0	
High BCT use (n≥10)	3	0.55 (0.23 to 0.87)	0	

a SMD: standard mean difference.

b Not applicable.

c BCT: behavior change technique.

### Meta-Analysis of Secondary Outcomes

Exergame-based behavior change interventions significantly increased MVPA (SMD 0.48, 95% CI 0.12-0.85, *I*^2^=65%) and step counts (SMD 0.54, 95% CI 0.13-0.94, *I*^2^=62%). However, the interventions did not show significant effects on LPA, MPA, VPA, SED, or BMI. Forest plots for these secondary outcomes are available in Multimedia Appendix 6.

## Discussion

### Main Findings

To our knowledge, this is the first meta-analysis of RCTs that evaluates the effects of exergame-based behavior change interventions for promoting PA and identifies the BCTs used in these interventions. This systematic review and meta-analysis included a total of 20 studies. Eight outcomes (ie, PA, MVPA, LPA, MPA, VPA, SED, step count, and BMI) were explored. The results demonstrated that exergame-based behavior change interventions significantly increased PA, MVPA, and step counts. Furthermore, subgroup analyses showed that intervention implementer (research assistants vs other implementers), game console (Xbox vs PlayStation vs Nintendo Wii), game participation type (individual game vs nonindividual game), measurement method (subjective vs objective), and number of BCTs (n<7 vs 7≤n<10 vs n≥10) significantly influenced the effectiveness of these interventions.

### Interpretation of the Findings of Meta-Analyses

In our study, the pooled SMD for PA was 0.19 (95% CI 0.05-0.33), indicating a small effect with statistical significance. This finding is partially consistent with a previous meta-analysis conducted by Moller et al [8] in 2023, which reported a moderate effect size (0.53, 95% CI 0.32-0.73) of exergame interventions on PA behaviors. The main difference between our study and that of Moller et al [8] is that our analysis was restricted to RCTs, whereas Moller’s study included both RCTs and other types of experimental studies. This inclusion may account for the slight differences between the results. However, compared to other study designs, RCTs provide stronger evidence. Overall, exergame-based interventions have shown effectiveness in promoting PA. Flow experience offers a potential explanation for the positive changes in PA behavior observed in exergame-based interventions [47]. Exergames represent an innovative way to combine exercise and gaming, enhancing participants’ pleasure and enjoyment during physical activities [48,49]. Therefore, participants are more likely to experience flow while engaging in exergames, leading to positive behavioral changes [50,51]. The relationship between positive behavior changes in PA and improved health outcomes merits further discussion. Children and adolescents are recommended to engage in at least an average of 60 minutes/day of MVPA, while adults should do a minimum of 150‐300 minutes of MPA, or 75‐150 minutes of VPA per week [2]. Given the variations among different PA metrics, total PA is not sufficient to infer changes in health outcomes resulting from PA. Therefore, we performed meta-analyses to examine the effects on various types of PA, including MVPA, LPA, MPA, VPA, and SED. Exergame-based interventions increased MVPA (SMD 0.48, 95% CI 0.12-0.85) and step counts (SMD 0.54, 95% CI 0.13-0.94), representing moderate effect sizes. However, effect sizes seen in different fields of research vary [52]. We recommend that future studies standardize the units of measurement for PA, such as minutes/day, to directly observe whether behavior change interventions lead to significant health improvements. The effects of exergame-based interventions on LPA, MPA, VPA, SED, and BMI were not statistically significant. Therefore, there is a need to explore better-designed exergame-based interventions.

### Interpretation of the Findings of Subgroup Analyses

RCTs included in the meta-analysis involved 2 types of participants: children and adults. Compared to adults, children exhibited greater improvements in PA behavior. This may be because exergame-based interventions are more attractive for children [53]. Exergames should be designed to meet the needs of various participant types to maximize their effectiveness [54,55]. Standardized design processes for exergame-based interventions can also be used in future studies [56]. Theory plays a crucial role in the design of interventions [57,58]. Only 3 RCTs used theory to guide exergame-based intervention design, and no significant subgroup differences in effect sizes were found between using and not using a theory. In the design of exergame-based interventions, the application of theory is not sufficiently emphasized, and theory-based interventions do not fully apply these theories.

The intervention site is also important in designing exergame-based interventions. In our study, we compared the effectiveness of interventions conducted in 3 different settings: school versus home versus hospital. Home-based interventions help participants overcome barriers associated with playing exergames, such as transportation and costs. In contrast, outside-home interventions (ie, school and hospital) are often supervised by professional staff, potentially enhancing the effectiveness of the intervention [59,60]. However, our study did not find significant differences between the 2 intervention sites, indicating that the choice of site may not be a main factor influencing the effectiveness of exergame-based interventions. Nevertheless, given the variability in the populations included in our study, the result should be treated with caution. We recommend that the selection of an intervention site should be based on the study population and research aims. Considering participants’ preferences regarding intervention sites is also important as it can influence their engagement and adherence to exergame-based interventions [61,62].

In order to achieve the recommended PA goals and transform behavior change interventions to significant health benefits, the intervention frequency and session duration should be designed and standardized. Although a slight difference in intervention frequency was observed, the sample sizes of included studies were limited, and our analysis failed to reveal significant differences between these subgroups, and the optimal intervention frequency and session duration require future exploration. The interventions performed by research assistants demonstrated significantly larger effect sizes compared to those implemented by other practitioners (eg, physical therapists). The reason behind this phenomenon might be that exergame-based interventions mainly require implementers to encourage and support participants in engaging with the interventions, rather than relying on specialized skills. Research assistants often have a higher sense of responsibility and motivation to adhere strictly to the study protocol, resulting in better intervention results.

Additionally, to our surprise, the game console brand acted as a significant factor in influencing health benefits effects. This may be related to the specific goals and development strategies used by the game developers in designing these games, particularly those aimed at maximizing either PA promotion or entertainment, depending on the focus of the game. According to our results, Xbox showed better PA promotion outcomes than PlayStation and Nintendo Wii. We advocate for collaborations between behavior change researchers and game companies to achieve the goal of both entertainment and health promotion. Nonindividual games (eg, team games) showed significantly better results than individual games, emphasizing the importance of integrating social support in intervention design, which is also one of the essential BCTs.

Another finding from our subgroup analysis is that interventions lasting 3 months or longer exhibited enhanced effectiveness, although no significant subgroup difference was observed. This may be explained by habit formation, which occurs when behavior is consistently repeated in a regular environment [63,64]. The more frequent the repetitions, the more likely it is that a behavior will become a habit. The link between habit formation and PA behavior change may be a valuable future research direction. The measurement of PA is important when it is the primary outcome of a study [65,66]. We have classified the measurement methods into subjective and objective measurements. Our results indicate that, compared to subjective methods, objective measurement demonstrated greater effectiveness in assessing PA. Using objective measurements in future studies is advised to enhance the reliability of the outcomes. Additionally, combining objective and subjective measurements can complement each other and provide rich result data.

### Interpretation of the Findings of BCTs

#### The Number of BCTs Used

We quantified the number of BCTs used in each study to explore whether it is an important influencing factor in exergame-based interventions. The subgroup analysis result is consistent with a previous study, finding that a greater number of BCTs (n≥10) correlated with increased adherence to PA [67]. However, this does not imply that studies using a larger number of BCTs will always achieve better outcomes. We advise that future researchers refer to the BCT taxonomy to guide their exergame-based intervention designs. The balance between the number of BCTs used and how they are effectively integrated is also important for intervention effectiveness.

#### Specific Most Frequently Used BCT

The most frequently used BCTs identified in our study were consistent with previous reviews, except for “12.5 adding objects to the environment,” which can be explained by the nature of exergame-based interventions incorporating game devices into scenes [23,24,68,69]. Other techniques, such as action planning, goal setting, self-monitoring, and giving instructions, are commonly used in promoting health behavior because they are simple and easy to implement. These techniques are recommended for use in future studies. However, this does not imply that other BCTs are less important. A broader range of less commonly used and more challenging BCTs (eg, habit formation) should also be explored in future studies.

### Strengths and Limitations

A comprehensive examination of the effectiveness of exergame-based interventions for promoting PA was conducted. Only RCTs were included in our systematic review and meta-analysis, ensuring the rigor of the study design. We explored 8 PA-related outcomes, providing a comprehensive overview of the results from exergame-based interventions. Additionally, subgroup analyses were performed to identify factors influencing effectiveness, offering valuable insights for the design of future interventions. However, the meta-analysis still has some limitations. First, although our systematic review and meta-analysis included 20 RCTs, only 11 of these, which provided total PA as an outcome, were included in subgroup analyses. The limited number of studies and sample sizes might restrict the ability to detect significant differences between subgroups, and the criteria for subgroup categorization were somewhat subjective, which might have overlooked other influencing factors due to classification issues. Therefore, the corresponding subgroup results should be treated with caution. Second, while the BCT coding was reviewed by another author (HMM) and showed good consistency, it still retained some degree of subjectivity. Additionally, differences in the reporting by the original study authors might have led to variations between the identified BCTs and those actually implemented in the interventions. Furthermore, the quality and intensity of the BCT used were more important than their labeling. For instance, both daily and weekly feedback were coded as “feedback” in the BCT taxonomy, but their effects were substantially different. This is why we do not conduct meta-regression in our study to identify effective BCTs. Although significant effects may be observed in some BCTs, the results were not reliable and could be misguiding. We recommend using BCT as a guide when designing interventions rather than inferring the effectiveness of using specific BCTs. Third, due to PA measurement differences across the original RCTs, we used SMD to aggregate effect sizes. However, this approach prevented direct comparisons with recommended PA dosage, thus limiting our ability to infer the health benefits from behavior changes. We suggest that standardized PA measurement methods should be used in future studies to enhance direct comparisons and interpretations.

### Conclusions

In conclusion, our systematic review and meta-analysis have demonstrated that exergame-based interventions are effective in promoting PA. Furthermore, we explored how different factors and BCTs influence the effectiveness of these interventions. Future trials are needed to further validate the insights proposed in our studies and to assess the long-term effects on PA.

## Supplementary material

10.2196/62906 Multimedia Appendix 1 Search strategy.

10.2196/62906 Multimedia Appendix 2 Behavior change technique (BCT) taxonomy.

10.2196/62906 Multimedia Appendix 3 Studies excluded at full-text review.

10.2196/62906 Multimedia Appendix 4 Characteristics of exergames.

10.2196/62906 Multimedia Appendix 5 Behavior change technique (BCT) identified in each study.

10.2196/62906 Multimedia Appendix 6 Forest plots of the secondary outcomes.

10.2196/62906 Checklist 1 PRISMA (Preferred Reporting Items for Systematic Reviews and Meta-Analyses) checklist.

## Abbreviations

BCT: behavior change technique
LPA: light physical activity
MeSH: Medical Subject Headings
MPA: moderate physical activity
MVPA: moderate to vigorous physical activity
PA: physical activity
PICOS: Participants, Interventions, Comparisons, Outcomes, and Study Design
PRISMA: Preferred Reporting Items for Systematic Reviews and Meta-Analyses
PROSPERO: International Prospective Register of Systematic Reviews
RCT: randomized controlled trial
SED: sedentary time
SMD: standard mean difference
VPA: vigorous physical activity
