# Supplementary material for: Exergame-Based Behavior Change Interventions for Promoting Physical Activity: Systematic Review and Meta-Analysis of Randomized Controlled Studies
Source: J Med Internet Res. 2025 Aug 8;27:e62906. doi: 10.2196/62906 (PMC12334110; doi:10.2196/62906)
Supplement: Multimedia Appendix 1 [file jmir-v27-e62906-s001.docx]

# Search strategy

## From inception to 2024/03/21

| PubMed | | Items found |
| --- | --- | --- |
| #1 | "exergaming"[MeSH Terms] OR "exergam*"[Title/Abstract] OR "active video gam*"[Title/Abstract] OR "virtual reality exercis*"[Title/Abstract] OR "Wii"[Title/Abstract] OR "Kinect"[Title/Abstract] OR "Nintendo"[Title/Abstract] OR "Xbox"[Title/Abstract] | 5,195 |
| #2 | "exercise"[MeSH Terms] OR "exercis*"[Title/Abstract] OR "physical activit*"[Title/Abstract] OR "physical exercis*"[Title/Abstract] OR "physical fitness"[Title/Abstract] OR "Sedentary"[Title/Abstract] OR "Inactivity"[Title/Abstract] OR "sport*"[Title/Abstract] | 690,366 |
| #3 | ("randomized controlled trial"[Publication Type] OR "randomized"[Title/Abstract] OR "placebo"[Title/Abstract]) | 1,053,180 |
| #4 | #1 AND #2 AND #3 | 646 |

| Embase | | Items found |
| --- | --- | --- |
| #1 | 'exergaming'/exp OR exergaming | 909 |
| #2 | 'exergam*':ab,ti OR 'active-video gam*':ab,ti OR 'virtual reality exercis*':ab,ti OR 'wii':ab,ti OR 'kinect':ab,ti OR 'nintendo':ab,ti OR 'xbox':ab,ti | 6,422 |
| #3 | #1 OR #2 | 6,568 |
| #4 | 'exercise'/exp OR exercise | 731,937 |
| #5 | 'exercis*':ab,ti OR 'physical activit*':ab,ti OR 'physical exercis*':ab,ti OR 'physical fitness':ab,ti OR 'sedentary':ab,ti OR 'inactivity':ab,ti OR 'sport*':ab,ti | 793,725 |
| #6 | #4 OR #5 | 1,004,657 |
| #7 | 'randomized controlled trial':ab,ti OR 'randomized':ab,ti OR 'rct':ab,ti OR 'placebo':ab,ti | 1,202,372 |
| #8 | #3 AND #6 AND #7 | 655 |

| Cochrane Library | | Items found |
| --- | --- | --- |
| #1 | (Exergaming):ab,ti,kw OR (Exergam*):ab,ti,kw OR (Active-Video Gam*):ab,ti,kw OR (Virtual Reality Exercis*):ab,ti,kw OR (Wii):ab,ti,kw OR (Kinect):ab,ti,kw OR (Nintendo):ab,ti,kw OR (Xbox):ab,ti,kw | 2,921 |
| #2 | (Exercise):ab,ti,kw OR (Exercis*):ab,ti,kw OR (Physical Activit*):ab,ti,kw OR (Physical exercis*):ab,ti,kw OR (Physical fitness):ab,ti,kw OR (Sedentary):ab,ti,kw OR (Inactivity):ab,ti,kw OR (Sport*):ab,ti,kw | 185,489 |
| #3 | (randomized controlled trial):ab,ti,kw OR (randomized):ab,ti,kw OR (RCT):ab,ti,kw OR (placebo):ab,ti,kw | 1,280,217 |
| #3 | #1 AND #2 AND #3 | 1,531 |

| Web of Science | | Items found |
| --- | --- | --- |
| #1 | TS=(Exergaming OR Exergam* OR Active-Video Gam* OR Virtual Reality Exercis* OR Wii OR Kinect OR Nintendo OR Xbox) | 16,969 |
| #2 | TS=(Exercise OR Exercis* OR Physical Activit* OR Physical exercis* OR Physical fitness OR Sedentary OR Inactivity OR Sport*) | 1,106,273 |
| #3 | TS= (randomized controlled trial OR randomized OR placebo OR RCT) | 1,226,581 |
| #4 | #1 AND #2 AND #3 | 1,292 |

| CINAHL | | Items found |
| --- | --- | --- |
| #1 | TI (Exergaming OR Exergam* OR Active-Video Gam* OR Virtual Reality Exercis* OR Wii OR Kinect OR Nintendo OR Xbox) OR AB (Exergaming OR Exergam* OR Active-Video Gam* OR Virtual Reality Exercis* OR Wii OR Kinect OR Nintendo OR Xbox) | 1,444 |
| #2 | TI (Exercise OR Exercis* OR Physical Activit* OR Physical exercis* OR Physical fitness OR Sedentary OR Inactivity OR Sport*) OR AB (Exercise OR Exercis* OR Physical Activit* OR Physical exercis* OR Physical fitness OR Sedentary OR Inactivity OR Sport*) | 178,296 |
| #3 | TI (randomized controlled trial or randomized or placebo or RCT) OR AB (randomized controlled trial or randomized or placebo or RCT) | 211,277 |
| #4 | #1 AND #2 AND #3 | 191 |

| SPORTDiscus | | Items found |
| --- | --- | --- |
| #1 | TI (Exergaming OR Exergam* OR Active-Video Gam* OR Virtual Reality Exercis* OR Wii OR Kinect OR Nintendo OR Xbox) OR AB (Exergaming OR Exergam* OR Active-Video Gam* OR Virtual Reality Exercis* OR Wii OR Kinect OR Nintendo OR Xbox) | 1,462 |
| #2 | TI (Exercise OR Exercis* OR Physical Activit* OR Physical exercis* OR Physical fitness OR Sedentary OR Inactivity OR Sport*) OR AB (Exercise OR Exercis* OR Physical Activit* OR Physical exercis* OR Physical fitness OR Sedentary OR Inactivity OR Sport*) | 537,996 |
| #3 | TI (randomized controlled trial or randomized or placebo or RCT) OR AB (randomized controlled trial or randomized or placebo or RCT) | 44,450 |
| #4 | #1 AND #2 AND #3 | 137 |
