# Supplementary material for: Exergame-Based Behavior Change Interventions for Promoting Physical Activity: Systematic Review and Meta-Analysis of Randomized Controlled Studies
Source: J Med Internet Res. 2025 Aug 8;27:e62906. doi: 10.2196/62906 (PMC12334110; doi:10.2196/62906)
Supplement: Multimedia Appendix 2 [file jmir-v27-e62906-s002.pdf]

BCT Taxonomy (v1): 93 hierarchically-clustered techniques

Page	Grouping and BCTs
**1**	**1. Goals and planning**
	1.1. Goal setting (behavior)
	1.2. Problem solving
	1.3. Goal setting (outcome)
	1.4. Action planning
	1.5. Review behavior goal(s)
	1.6. Discrepancy between current behavior and goal
	1.7. Review outcome goal(s)
	1.8. Behavioral contract
	1.9. Commitment
**3**	**2. Feedback and monitoring**
	2.1. Monitoring of behavior by others without feedback
	2.2. Feedback on behaviour
	2.3. Self-monitoring of behaviour
	2.4. Self-monitoring of outcome(s) of behaviour
	2.5. Monitoring of outcome(s) of behavior without feedback
	2.6. Biofeedback
	2.7. Feedback on outcome(s) of behavior
**5**	**3. Social support**
	3.1. Social support (unspecified)
	3.2. Social support (practical)
	3.3. Social support (emotional)
**6**	**4. Shaping knowledge**
	4.1. Instruction on how to perform the behavior
	4.2. Information about Antecedents
	4.3. Re-attribution
	4.4. Behavioral experiments
**7**	**5. Natural consequences**
	5.1. Information about health consequences
	5.2. Salience of consequences
	5.3. Information about social and environmental consequences
	5.4. Monitoring of emotional consequences
	5.5. Anticipated regret
	5.6. Information about emotional consequences
**8**	**6. Comparison of behaviour**
	6.1. Demonstration of the behavior
	6.2. Social comparison
	6.3. Information about others’ approval
**9**	**7. Associations**
	7.1. Prompts/cues
	7.2. Cue signalling reward
	7.3. Reduce prompts/cues
	7.4. Remove access to the reward
	7.5. Remove aversive stimulus
	7.6. Satiation
	7.7. Exposure
	7.8. Associative learning
**10**	**8. Repetition and substitution**
	8.1. Behavioral practice/rehearsal
	8.2. Behavior substitution
	8.3. Habit formation
	8.4. Habit reversal
	8.5. Overcorrection
	8.6. Generalisation of target behavior
	8.7. Graded tasks
**11**	**9. Comparison of outcomes**
	9.1. Credible source
	9.2. Pros and cons
	9.3. Comparative imagining of future outcomes
**12**	**10. Reward and threat**
	10.1. Material incentive (behavior)
	10.2. Material reward (behavior)
	10.3. Non-specific reward
	10.4. Social reward
	10.5. Social incentive
	10.6. Non-specific incentive
	10.7. Self-incentive
	10.8. Incentive (outcome)
	10.9. Self-reward
	10.10. Reward (outcome)
	10.11. Future punishment
**15**	**11. Regulation**
	11.1. Pharmacological support
	11.2. Reduce negative emotions
	11.3. Conserving mental resources
	11.4. Paradoxical instructions
**16**	**12. Antecedents**
	12.1. Restructuring the physical environment
	12.2. Restructuring the social environment
	12.3. Avoidance/reducing exposure to cues for the behavior
	12.4. Distraction
	12.5. Adding objects to the environment
	12.6. Body changes
**17**	**13. Identity**
	13.1. Identification of self as role model
	13.2. Framing/reframing
	13.3. Incompatible beliefs
	13.4. Valued self-identify
	13.5. Identity associated with changed behavior
**18**	**14. Scheduled consequences**
	14.1. Behavior cost
	14.2. Punishment
	14.3. Remove reward
	14.4. Reward approximation
	14.5. Rewarding completion
	14.6. Situation-specific reward
	14.7. Reward incompatible behavior
	14.8. Reward alternative behavior
	14.9. Reduce reward frequency
	14.10. Remove punishment
**19**	**15. Self-belief**
	15.1. Verbal persuasion about capability
	15.2. Mental rehearsal of successful performance
	15.3. Focus on past success
	15.4. Self-talk
**19**	**16. Covert learning**
	16.1. Imaginary punishment
	16.2. Imaginary reward
	16.3. Vicarious consequences
