# Supplementary material for: Exergame-Based Behavior Change Interventions for Promoting Physical Activity: Systematic Review and Meta-Analysis of Randomized Controlled Studies
Source: J Med Internet Res. 2025 Aug 8;27:e62906. doi: 10.2196/62906 (PMC12334110; doi:10.2196/62906)
Supplement: Multimedia Appendix 3 [file jmir-v27-e62906-s003.docx]

| Studies excluded at full-text review | | |
| --- | --- | --- |
| Number | Reference | Reason |
| 1 | Physical Activity and the Nintendo Wii: a Psycho-Physiological Approach. Sport journal. 2014 2014:1‐. PMID: rayyan-723582870. | Lack of outcome data |
| 2 | Mii-vitaliSe: a pilot randomised controlled trial of a home gaming system (Nintendo Wii) to increase activity levels, vitality and well-being in people with multiple sclerosis. BMJ open. 2017 2017;7:e016966. PMID: rayyan-723582872. | Lack of outcome data |
| 3 | Al-Ali FS, Zhou H, Hamad A, Ibrahim RA, Talal T, Najafi B. Exercise games to improve balance and mobility in diabetic patients undergoing hemodialysis: a randomized controlled trial. Journal of the American Society of Nephrology : JASN. 2018 2018;29:321. PMID: rayyan-723582943. | Wrong outcome |
| 4 | Barry G, van Schaik P, MacSween A, Dixon J, Martin D. Exergaming (XBOX Kinect™) versus traditional gym-based exercise for postural control, flow and technology acceptance in healthy adults: a randomised controlled trial. BMC sports science, medicine and rehabilitation. 2016 2016;8(1). PMID: rayyan-723583052. doi:10.1186/s13102-016-0050-0. | Wrong outcome |
| 5 | Basha MA, Aboelnour NH, Aly SM, Kamel FAH. Impact of Kinect-based virtual reality training on physical fitness and quality of life in severely burned children: a monocentric randomized controlled trial. Annals of physical and rehabilitation medicine. 2022 2022;65(1):101471. PMID: rayyan-723583058. doi:10.1016/j.rehab.2020.101471. | Wrong outcome |
| 6 | Birgit Boehm B, Boehm H, Oberhoffer R, Bauer CP. Active video games for physical activity promotion in obese youth: Possibilities and risks. European Journal of Preventive Cardiology. 2014 2014;21(1):S148. PMID: rayyan-723583103. doi:10.1177/2047487314534585. | Conference abstract |
| 7 | Bondoc S, Hewitt P, Frey N, McQuide B, Johnson A. The effect of wii-based interventions on physical, cognitive and social functioning among pre-frail elderly persons. Archives of physical medicine and rehabilitation. 2011 2011;92(10):1700. PMID: rayyan-723583114. | Conference abstract |
| 8 | Cebolla iMA, Álvarez-Pitti JC, Guixeres Provinciale J, Lisón JF, Baños Rivera R. Alternative options for prescribing physical activity among obese children and adolescents: brisk walking supported by an exergaming platform. Nutricion hospitalaria. 2014 2014;31(2):841‐8. PMID: rayyan-723583223. doi:10.3305/nh.2015.31.2.7929. | Wrong outcome |
| 9 | Comeras-Chueca C, Villalba-Heredia L, Perez-Llera M, Lozano-Berges G, Marin-Puyalto J, Vicente-Rodriguez G, et al. Assessment of Active Video Games' Energy Expenditure in Children with Overweight and Obesity and Differences by Gender. International journal of environmental research and public health. 2020 2020;17(18):1‐17. PMID: rayyan-723583321. doi:10.3390/ijerph17186714. | Wrong outcome |
| 10 | Da Silva TF, De Franca ACL, De Souza MF, Sergio Silva A. A SINGLE SESSION OF ACTIVE VIDEO GAME PLAY PROMOTES POST-EXERCISE HYPOTENSION IN HYPERTENSIVE MIDDLE-AGED SUBJECTS. Human Movement. 2018 2018;19(2):82-9. PMID: rayyan-723583364. doi:10.5114/hm.2018.74063. | Wrong outcome |
| 11 | Ditchburn JL, Van Schaik P, Dixon J, MacSween A, Martin D. The effects of exergaming on pain, postural control, technology acceptance and flow experience in older people with chronic musculoskeletal pain: a randomised controlled trial. BMC sports science, medicine and rehabilitation. 2020 2020;12(1). PMID: rayyan-723583429. doi:10.1186/s13102-020-00211-x. | Wrong outcome |
| 12 | Ferraris C, Pepe NL, Trentani C, Tinelli S, Borrelli P, Vandoni M, et al. ENJOY project-exergames & healthy nutrition joined against obesity for one year: Three-month outcome. International Journal of Sport Nutrition and Exercise Metabolism. 2015 2015;25:9. PMID: rayyan-723583516. doi:10.1123/ijsnem.25.s1.s1. | Conference abstract |
| 13 | Fitzgerald D, Trakarnratanakul N, Smyth B, Caulfield B. Effects of a wobble board-based therapeutic exergaming system for balance training on dynamic postural stability and intrinsic motivation levels. Journal of orthopaedic and sports physical therapy. 2010 2010;40(1):11‐9. PMID: rayyan-723583533. doi:10.2519/jospt.2010.3121. | Wrong outcome |
| 14 | Gao Z, Zeng N, Pope ZC, Wang R, Yu F. Effects of exergaming on motor skill competence, perceived competence, and physical activity in preschool children. J Sport Health Sci. 2019 Mar;8(2):106-13. PMID: 30997256. doi: 10.1016/j.jshs.2018.12.001. | Wrong study design |
| 15 | García-Bravo S, Cano-de-la-Cuerda R, Domínguez-Paniagua J, Campuzano-Ruiz R, Barreñada-Copete E, López-Navas MJ, et al. Effects of Virtual Reality on Cardiac Rehabilitation Programs for Ischemic Heart Disease: a Randomized Pilot Clinical Trial. International journal of environmental research and public health. 2020 2020;17(22). PMID: rayyan-723583589. doi:10.3390/ijerph17228472. | Wrong outcome |
| 16 | Garde A, Chowdhury M, Rollinson AU, Johnson M, Prescod P, Chanoine JP, et al. A Multi-Week Assessment of a Mobile Exergame Intervention in an Elementary School. Games for health journal. 2018 2018;7(1):1‐8. PMID: rayyan-723583602. doi:10.1089/g4h.2017.0023. | Wrong outcome |
| 17 | Gribbon A, McNeil J, Jay O, Tremblay MS, Chaput JP. Active video games and energy balance in male adolescents: a randomized crossover trial. American journal of clinical nutrition. 2015 2015;101(6):1126‐34. PMID: rayyan-723583673. doi:10.3945/ajcn.114.105528. | Wrong outcome |
| 18 | Hauer K, Litz E, Gunther-Lange M, Ball C, de Bruin ED, Werner C. Effectiveness and sustainability of a motor-cognitive stepping exergame training on stepping performance in older adults: a randomized controlled trial. European review of aging and physical activity. 2020 2020;17:17. PMID: rayyan-723583759. doi:10.1186/s11556-020-00248-4. | Wrong outcome |
| 19 | Hsia SH, Magliano LA, Sanchez H, Storer TW. “Dance dance revolution” exergaming vs. treadmill exercise in type 2 diabetes. Diabetes. 2013 2013;62:A186‐A7. PMID: rayyan-723583820. doi:10.2337/db13-680-858. | Conference abstract |
| 20 | Huang HC, Van Nguyen H, Cheng TCE, Wong MK, Chiu HY, Yang YH, et al. A Randomized Controlled Trial on the Role of Enthusiasm About Exergames Players' Perceptions of Exercise. Games for Health Journal. 2019 2019-6;8(3):220-6. PMID: rayyan-723583835. doi:10.1089/g4h.2018.0057. | Wrong outcome |
| 21 | Irwin BC, Feltz DL, Kerr NL. Silence is golden: effect of encouragement in motivating the weak link in an online exercise video game. J Med Internet Res. 2013 2013-6-4;15(6):e104. PMID: rayyan-723583863. doi:10.2196/jmir.2551. | Wrong outcome |
| 22 | Jaarsma T, Klompstra L, Ben Gal T, Ben Avraham B, Boyne J, Bäck M, et al. Effects of exergaming on exercise capacity in patients with heart failure: results of an international multicentre randomized controlled trial. European Journal of Heart Failure. 2021 2021;23(1):114-24. PMID: rayyan-723583873. doi:10.1002/ejhf.1754. | Wrong outcome |
| 23 | Jaber M, Farr W, Morris C, Bremmer S, Male I, Green D. Barriers and facilitators to physical activity participation and engagement in Wii-Fit hometherapy programmes for children with cerebral palsy. Developmental medicine and child neurology. 2017 2017;59:22. PMID: rayyan-723583879. doi:10.1111/dmcn.13455. | Conference abstract |
| 24 | Jendro AM, Padala PR, Gauss CH, Garrison MK, Wilson KB, Parkes CM, et al. Does Participation In An Exercise Program Increase Physical Activity On Non-Exercise Days? Medicine & Science in Sports & Exercise. 2021 2021;53(8):265-. PMID: rayyan-723583888. doi:10.1249/01.mss.0000762172.62045.bc. | Conference abstract |
| 25 | Kauhanen L, Järvelä L, Lähteenmäki PM, Arola M, Heinonen OJ, Axelin A, et al. Active video games to promote physical activity in children with cancer: a randomized clinical trial with follow-up. BMC Pediatrics. 2014 2014;14(1):94-. PMID: rayyan-723583967. doi:10.1186/1471-2431-14-94. | Protocol |
| 26 | Klompstra L, Jaarsma T, Piepoli MF, Ben Gal T, Evangelista L, Strömberg A, et al. Objectively measured physical activity in patients with heart failure: a sub-analysis from the HF-Wii study. Eur J Cardiovasc Nurs. 2022 Jun 30;21(5):499-508. PMID: 34993536. doi: 10.1093/eurjcn/zvab133. | Wrong outcome |
| 27 | Klompstra L, Jaarsma T, Strömberg A. Exergaming to increase the exercise capacity and daily physical activity in heart failure patients: a pilot study. Bmc Geriatrics. 2014 2014-11;14. PMID: rayyan-723584043. doi:10.1186/1471-2318-14-119. | Wrong outcome |
| 28 | Lau WC, Zhang S, Maddison R. The effect of a school-based active video game intervention on children's aerobic fitness, physical activity level, and exercise related psychological variables: a preliminary RCT trial. Obesity facts. 2015 2015;8:145. PMID: rayyan-723584104. doi:10.1159/000382140. | Conference abstract |
| 29 | Lee HK, Jin J. The effect of a virtual reality exergame on motor skills and physical activity levels of children with a developmental disability. Research in developmental disabilities. 2023 2023;132:104386. PMID: rayyan-723584118. doi:10.1016/j.ridd.2022.104386. | Wrong outcome |
| 30 | LeGear T, LeGear M, Preradovic D, Wilson G, Kirkham A, Camp PG. Does a Nintendo Wii exercise program provide similar exercise demands as a traditional pulmonary rehabilitation program in adults with COPD? Clinical respiratory journal. 2016 2016;10(3):303‐10. PMID: rayyan-723584137. doi:10.1111/crj.12216. | Wrong outcome |
| 31 | Lin XY, Zhang L, Yoon S, Zhang RY, Lachman ME. A Social Exergame Intervention to Promote Physical Activity, Social Support, and Well-Being in Family Caregivers. Gerontologist. 2023 2023-10;63(9):1456-66. PMID: rayyan-723584187. doi:10.1093/geront/gnad028. | Wrong comparison |
| 32 | Lu AS, Pelarski V, Alon D, Baran A, McGarrity E, Swaminathan N, et al. The effect of narrative element incorporation on physical activity and game experience in active and sedentary virtual reality games. Virtual Real. 2023 2023-1-31:1-16. PMID: rayyan-723584223. doi:10.1007/s10055-023-00754-7. | Wrong comparison |
| 33 | Lyons EJ, Tate DF, Komoski SE, Carr PM, Ward DS. Novel approaches to obesity prevention: effects of game enjoyment and game type on energy expenditure in active video games. J Diabetes Sci Technol. 2012 2012-7-1;6(4):839-48. PMID: rayyan-723584236. doi:10.1177/193229681200600415. | Wrong comparison |
| 34 | Maddison R, Foley L, Jiang Y, Ni Mhurchu C, Jull A, Rodgers A, et al. Electronic games to aid motivation to exercise: a randomized controlled trial. Obesity reviews. 2010 2010;11:50. PMID: rayyan-723584247. doi:10.1111/j.1467-789X.2010.00763-4.x. | Conference abstract |
| 35 | Maddison, R. et al. Effects of active video games on body composition: a randomized controlled trial. American Journal of Clinical Nutrition 94, 156-163 (2011). https://doi.org:doi:10.3945/ajcn.110.009142 | Wrong outcome |
| 36 | Maddison R, Foley L, Ni Mhurchu C, Jull A, Jiang Y, Prapavessis H, et al. Feasibility, design and conduct of a pragmatic randomized controlled trial to reduce overweight and obesity in children: The electronic games to aid motivation to exercise (eGAME) study. BMC Public Health. 2009 2009;9. PMID: rayyan-723584253. doi:10.1186/1471-2458-9-146. | Protocol |
| 37 | Masoud AE, Shaheen AAM, Algabbani MF, AlEisa E, AlKofide A. Effectiveness of exergaming in reducing cancer-related fatigue among children with acute lymphoblastic leukemia: a randomized controlled trial. Annals of medicine. 2023 2023;55(1):2224048. PMID: rayyan-723584309. doi: doi:10.1080/07853890.2023.2224048. | Wrong outcome |
| 38 | Monedero J, McDonnell AC, Keoghan M, O'Gorman DJ. Modified Active Videogame Play Results in Moderate-Intensity Exercise. Games for Health Journal. 2014 2014-8;3(4):234-40. PMID: rayyan-723584397. doi:10.1089/g4h.2013.0096. | Wrong outcome |
| 39 | Monedero J, Murphy EE, O'Gorman DJ. Energy expenditure and affect responses to different types of active video game and exercise. PloS one. 2017 2017;12(5):e0176213. PMID: rayyan-723584398. doi:10.1371/journal.pone.0176213. | Wrong outcome |
| 40 | Ni Mhurchu C, Maddison R, Jiang Y, Jull A, Prapavessis H, Rodgers A. Couch potatoes to jumping beans: a pilot study of the effect of active video games on physical activity in children. International journal of behavioral nutrition and physical activity. 2008 2008;5. PMID: rayyan-723584720. doi:10.1186/1479-5868-5-8. | Wrong outcome |
| 41 | Oesch P, Kool J, Fernandez-Luque L, Brox E, Evertsen G, Civit A, et al. Exergames versus self-regulated exercises with instruction leaflets to improve adherence during geriatric rehabilitation: a randomized controlled trial. BMC Geriatrics. 2017 2017;17:1-9. PMID: rayyan-723584742. doi:10.1186/s12877-017-0467-7. | Wrong outcome |
| 42 | Patricio DS, Aires YR, Medeiros CM. Gamification: A Tool to Increase the Frequency of Physical Activity in Overweight Adolescents. Journal of Exercise Physiology Online. 2020 2020;23(3):13-24. PMID: rayyan-723584816. | Wrong outcome |
| 43 | Peng W, Pfeiffer KA, Winn B, Lin JH, Suton D. A pilot randomized, controlled trial of an active video game physical activity intervention. Health psychology. 2015 2015;34:1229‐39. PMID: rayyan-723584831. doi:10.1037/hea0000302. | Wrong outcome |
| 44 | Perrin T, Faure C, Nay K, Cattozzo G, Sorel A, Kulpa R, et al. Virtual Reality Gaming Elevates Heart Rate but Not Energy Expenditure Compared to Conventional Exercise in Adult Males. International journal of environmental research and public health. 2019 2019;16(22). PMID: rayyan-723584839. doi:10.3390/ijerph16224406. | Wrong outcome |
| 45 | Rhodes RE, Blanchard CM, Bredin SSD, Beauchamp MR, Maddison R, Warburton DER. Stationary cycling exergame use among inactive children in the family home: a randomized trial. Journal of behavioral medicine. 2017 2017;40(6):978‐88. PMID: rayyan-723584946. doi:10.1007/s10865-017-9866-7. | Wrong outcome |
| 46 | Roglin L, Ketelhut S, Ketelhut K, Kircher E, Ketelhut RG, Martin-Niedecken AL, et al. Adaptive High-Intensity Exergaming: the More Enjoyable Alternative to Conventional Training Approaches Despite Working Harder. Games for health journal. 2021 2021;10(6):400‐7. PMID: rayyan-723584987. doi:10.1089/g4h.2021.0014. | Wrong outcome |
| 47 | Roure C, Pasco D, Benoit N, Deldicque L. Impact of a Design-Based Bike Exergame on Young Adults' Physical Activity Metrics and Situational Interest. Research quarterly for exercise and sport. 2020 2020;91(2):309‐15. PMID: rayyan-723585001. doi: 10.1080/02701367.2019.1665621. | Wrong outcome |
| 48 | Ruivo J, Karim K, OʼShea R, Oliveira RCS, Keary L, OʼBrien C, et al. In-class Active Video Game Supplementation and Adherence to Cardiac Rehabilitation. J Cardiopulm Rehabil Prev. 2017 2017-7;37(4):274-8. PMID: rayyan-723585009. doi: 10.1097/hcr.0000000000000224. | Conference abstract |
| 49 | Sabel M, Broeren J, Arvidsson D, Sjölund A, Gillenstrand J, Ljungberg C, et al. Physical activity through home-based exercise-gaming after childhood brain tumour treatment-a feasability study. Pediatric Blood and Cancer. 2013 2013;60:165. PMID: rayyan-723585027. doi:10.1002/pbc.24719. | Conference abstract |
| 50 | Sabel M, Sjölund A, Broeren J, Arvidsson D, Saury JM, Blomgren K, et al. Active video gaming improves body coordination in survivors of childhood brain tumours. Disability and rehabilitation. 2016 2016;38(21):2073‐84. PMID: rayyan-723585029. doi:10.3109/09638288.2015.1116619. | Wrong outcome |
| 51 | Schumacher H, Stuwe S, Kropp P, Diedrich D, Freitag S, Greger N, et al. A prospective, randomized evaluation of the feasibility of exergaming on patients undergoing hematopoietic stem cell transplantation. Bone marrow transplantation. 2018 2018;53(5):584‐90. PMID: rayyan-723585111. doi:10.1038/s41409-017-0070-8. | Wrong outcome |
| 52 | Serber ER, Ciccolo J, Palmer K, Cobb V, Tilkemeier PL, Bock BC. The feasibility of exercise videogames for cardiovascular risk reduction among adults: a pilot for "Wii heart fitness". J Sports Med Phys Fitness. 2016 2016-3;56(3):319-27. PMID: rayyan-723585137. | Lack of outcome data |
| 53 | Simons M, Brug J, Chinapaw MJ, de Boer M, Seidell J, de Vet E. Replacing non-active video gaming by active video gaming to prevent excessive weight gain in adolescents. PloS one. 2015 2015;10(7):e0126023. PMID: rayyan-723585170. doi:10.1371/journal.pone.0126023. | Wrong outcome |
| 54 | Smith W, Moran RA, Fu CA, Scott TA. Active video gaming compared to treadmill walking and TV watching in obese children and adolescents. FASEB journal. 2017 2017;31(1). PMID: rayyan-723585180. | Conference abstract |
| 55 | Sonbahar-Ulu H, Inal-Ince D, Saglam M, Cakmak A, Vardar-Yagli N, Calik-Kutukcu E, et al. Active video gaming in primary ciliary dyskinesia: a randomized controlled trial. European journal of pediatrics. 2022 2022;181(8):2891‐900. PMID: rayyan-723585191. doi:10.1007/s00431-022-04490-z. | Wrong outcome |
| 56 | Staiano AE, Beyl RA, Hsia DS, Katzmarzyk PT, Newton RL. Twelve weeks of dance exergaming in overweight and obese adolescent girls: transfer effects on physical activity, screen time, and self-efficacy. Journal of sport and health science. 2017 2017;6(1):4‐10. PMID: rayyan-723585211. doi:10.1016/j.jshs.2016.11.005. | Wrong outcome |
| 57 | Straker L, Howie E, Abbott R, Smith A. Active video games: are they an effective approach to reducing sedentary time and increasing physical activity in children? Journal of science and medicine in sport. 2014 2014;18:e65. PMID: rayyan-723585224. doi:10.1016/j.jsams.2014.11.292. | Conference abstract |
| 58 | Ufholz K, Flack KD, Johnson L, Roemmich JN. Active Videogames to Promote Traditional Active Play: Increasing the Reinforcing Value of Active Play Among Low-Active Children. Games Health J. 2019 Dec 3. PMID: 31800323. doi:10.1089/g4h.2019.0040. | Wrong outcome |
| 59 | Ufholz KE, Flack KD, Roemmich JN. The influence of active video game play upon physical activity and screen-based activities in sedentary children. PloS one. 2022 2022;17(6):e0269057. PMID: rayyan-723585346. doi:10.1371/journal.pone.0269057. | Wrong study design |
| 60 | Vallabhajosula S, Holder JB, Bailey EK. Effect of Exergaming on Physiological Response and Enjoyment During Recess in Elementary School-Aged Children: A Pilot Study. Games Health J. 2016 2016-10;5(5):325-32. PMID: rayyan-723585364. doi:10.1089/g4h.2016.0032. | Lack of outcome data |
| 61 | van Santen J, Meiland FJM, Dröes RM, van Straten A, Bosmans JE. Cost‐effectiveness of exergaming compared to regular day‐care activities in dementia: results of a randomised controlled trial in The Netherlands. Health & social care in the community. 2022 2022;30(5):e1794‐e804. PMID: rayyan-723585392. doi:10.1111/hsc.13608. | Wrong outcome |
| 62 | Xian Y, Kakinami L, Peterson ED, Mustian KM, Fernandez ID. Will Nintendo "Wii Fit" Get You Fit? An Evaluation of the Energy Expenditure from Active-Play Videogames. Games Health J. 2014 2014-4;3(2):86-91. PMID: rayyan-723585504. doi:10.1089/g4h.2013.0078. | Wrong outcome |
| 63 | Zadro JR, Shirley D, Simic M, Mousavi SJ, Ceprnja D, Maka K, et al. Video-Game–Based Exercises for Older People With Chronic Low Back Pain: A Randomized Controlledtable Trial (GAMEBACK). Physical Therapy. 2019 2019;99(1):14-27. PMID: rayyan-723585559. doi:10.1093/ptj/pzy112. | Wrong outcome |
| 64 | Zhou H, Halder D, Hamad A, Al-Ali F, Talal T, Ibrahim R, et al. Game-based non-weight bearing exercise to improve daily physical activity in diabetic patients undergoing hemodialysis: a pilot randomized controlled trial. Journal of diabetes science and technology. 2018 2018;12(2):A100. PMID: rayyan-723585587. doi:10.1177/1932296818761742. | Conference abstract |
| 65 | Cutter CJ, Schottenfeld RS, Moore BA, Ball SA, Beitel M, Savant JD, et al. A pilot trial of a videogame-based exercise program for methadone maintained patients. Journal of substance abuse treatment. 2014 2014;47(4):299‐305. PMID: rayyan-723583343. doi:10.1016/j.jsat.2014.05.007. | Wrong outcome |
