# Supplementary material for: Exergame-Based Behavior Change Interventions for Promoting Physical Activity: Systematic Review and Meta-Analysis of Randomized Controlled Studies
Source: J Med Internet Res. 2025 Aug 8;27:e62906. doi: 10.2196/62906 (PMC12334110; doi:10.2196/62906)
Supplement: Multimedia Appendix 4 [file jmir-v27-e62906-s004.docx]

# Characteristics of exergames

| No. | Reference | Game name | Game primary device | Game content |
| --- | --- | --- | --- | --- |
| 1 | Adamo et al [34] | GameBike | Sony play station 2 | cycling |
| 2 | Baranowski et al [35] | Active Life-Extreme Challenge, EA Sports Active, Dance Dance Revolution, Wii Fit Plus, Wii Sports | Nintendo Wii | sports, dance |
| 3 | Campelo et al [15] | Island Cycling, (Free) Basic Run, Free Steps, Basic Steps, (Super) Hula Hoop, Lunge, Side Lunge, Torso Twist, Rowing Squat, Table Tilt, Tilt City, Trampoline Target, Bird's Eye Bull's Eye, Ski Slalom, Sun Salutation, Chair, Half Moon | Nintendo Wii | aerobic, strength, balance, yoga |
| 4 | Cavusoglu et al [16] | Lunge, Sideways leg lift, Single-leg extension, Sun salutation, Jogging, Tree, Rhythm parade, Step, Rowing squat | Nintendo Wii | aerobic, strength, yoga |
| 5 | Comeras-Chueca et al [13] | Kinect Adventures, Kinect Sports, Wii Sports, Just Dance, Mario and Sonic at the Olympic Games, Dance Dance Revolution, Mario and Sonic at the Olympic Games, BKOOL Interactive Cycling | Xbox 360, Nintendo Wii, BKOOL Interactive Cycling Simulator | adventures, sports, dance, cycling |
| 6 | Garde et al [37] | MobileKids Monster Manor | Phone | walk |
| 7 | Howie et al [38] | Kinect Adventures, Start the Party, Eye Pet, Sports Champions | Sony Play Station 3, Xbox 360 | adventure, sports, mini-games, pet simulation |
| 8 | Lau et al [40] | Xbox Sport Season Series 1 and 2 | Xbox 360 | sports |
| 9 | Sousa et al [17] | Beat Saber | HTC Vive Pro System (virtual reality headset) | rhythms |
| 10 | Swartz et al [18] | Just Dance, Zumba, Wii Fit U minigames, Your Shape Fitness Evolved 2012 | Nintendo Wii, Xbox 360 | walking, yoga, dance, rhythms, zen energy |
| 11 | Trost et al [45] | Kinect Adventures, Kinect Sports | Xbox | adventures, sports |
| 12 | van Santen et al [46] | Interactive Cycling | DiFiets, Fietslabyrint, PraxFit, or SilverFit Mile. | cycling |
| 13 | Maloney et al [42] | Dance Dance Revolution | Sony play station 2 | dance |
| 14 | Maloney et al [43] | Dance Dance Revolution | Sony play station 2 | dance |
| 15 | Şimşek and Çekok [44] | Wii Sports, Wii Fit | Nintendo Wii | yoga, strength training, balance, aerobic exercise |
| 16 | Bowling et al [14] | Just Dance 3, Shape Up 3, Kinect Sports Season 2 | Xbox | dance, sports |
| 17 | Kempf and Martin [39] | Wii Fit Plus | Nintendo Wii | yoga, strength training, balance, aerobic exercise |
| 18 | Lwin and Malik [41] | Dance Dance Revolution, Wii tennis, Wii boxing | Nintendo Wii | dance, sports |
| 19 | Cowdery et al [36] | Zombies, Run!, The Walk | smartphone | adventure |
| 20 | Hamari et al [12] | NA | Nintendo Wii | NA |

Abbreviation: NA, not applicable.

| No. | Game name | Description |
| --- | --- | --- |
| 1 | GameBike | The GameBike, developed by Cat Eye Electronics Ltd., is an interactive video gaming system integrated with a Sony PlayStation 2 and a 42" flat screen television monitor. It features a handlebar-mounted game controller, allowing participants to play most race-based video games on the PlayStation 2 while cycling. The GameBike tracks the user's speed based on cycling cadence, with faster pedaling resulting in faster movement in the virtual game world. Users can adjust the bike's resistance and choose the intensity and duration of their exercise sessions, making it a versatile and engaging fitness tool. |
| 2 | Active Life-Extreme Challenge | Active Life: Extreme Challenge, developed for the Nintendo Wii by Bandai Namco, is an action-packed sports game that uses the special Active Life mat controller. Players engage in various extreme sports activities like surfing, skateboarding, and rock climbing by performing physical movements on the mat. |
| 3 | EA Sports Active | EA Sports Active, developed for the Nintendo Wii by Electronic Arts, is a fitness game designed to provide a comprehensive workout experience. It includes a variety of exercises such as cardio, strength training, and flexibility routines, using accessories like a leg strap and resistance band. |
| 4 | Dance Dance Revolution | Dance Dance Revolution (DDR), developed by Konami, is a rhythm and dance game available on multiple platforms, including Nintendo Wii and PlayStation. Players stand on a dance mat and follow on-screen arrows by stepping on corresponding directions in time with the music. |
| 5 | Wii Fit Plus | Wii Fit Plus, developed by Nintendo for the Nintendo Wii, is a fitness game that uses the Wii Balance Board. It includes a variety of exercises such as yoga, strength training, aerobics, and balance games. |
| 6 | Wii Sports | Wii Sports, developed by Nintendo for the Nintendo Wii, is a sports simulation game that includes five sports: tennis, baseball, bowling, golf, and boxing. Using the Wii Remote's motion-sensing capabilities, players perform real-life movements to control the game, providing an engaging and intuitive gaming experience suitable for all ages. |
| 7 | Island Cycling | Island Cycling is an exergame where players simulate cycling on a virtual island. |
| 8 | (Free) Basic Run | (Free) Basic Run is an exergame that simulates running in a virtual environment. |
| 9 | Free Steps | Free Steps is an exergame that allows players to perform step exercises at their own pace. |
| 10 | Basic Steps | Basic Steps is an exergame that guides players through fundamental step exercises. |
| 11 | (Super) Hula Hoop | (Super) Hula Hoop is an exergame where players simulate hula hooping by moving their hips in rhythm with the game. |
| 12 | Lunge | Lunge is an exergame exercise that guides players through lunges to strengthen the lower body. |
| 13 | Side Lunge | Side Lunge is an exergame exercise that involves performing lateral lunges. |
| 14 | Torso Twist | Torso Twist is an exergame exercise where players perform twisting motions with their torso. |
| 15 | Rowing Squat | Rowing Squat is an exergame exercise that combines rowing motions with squats. |
| 16 | Table Tilt | Table Tilt is an exergame that challenges players to tilt a virtual table to guide balls into targets. |
| 17 | Tilt City | Tilt City is an exergame where players tilt their bodies to navigate objects through a virtual city. |
| 18 | Trampoline Target | Trampoline Target is an exergame where players simulate jumping on a trampoline to hit virtual targets. |
| 19 | Bird's Eye Bull's Eye | Bird's Eye Bull's Eye is an exergame where players aim and hit targets from a top-down perspective. |
| 20 | Ski Slalom | Ski Slalom is an exergame where players simulate skiing down a virtual slope, navigating through a series of gates. |
| 21 | Sun Salutation | Sun Salutation is an exergame that guides players through a series of yoga poses known as the Sun Salutation. |
| 22 | Chair | Chair is an exergame exercise that guides players through the yoga pose known as Chair Pose. |
| 23 | Half Moon | Half Moon is an exergame that guides players through the Half Moon yoga pose. |
| 24 | Sideways leg lift | While the child is on the balance board and with one foot in the air, she maintains her balance and makes abduction movements with her foot in the air and the arm on the opposite side at the same time. |
| 25 | Single-leg extension | The child stands with one leg slightly extended on the balance board. With the sign, the arm on the same side as the extended leg flexes, the leg goes into extension, then returns to the starting position. |
| 26 | Jogging | The child walks on the normal floor for 3-5 minutes at 60% of the constant walking speed while the Nintendo Wii controller is in his hand or in his pocket. |
| 27 | Tree | The child takes a deep breath and exhales while keeping his balance, with one foot on the balance board and the other foot in the air. |
| 28 | Rhythm parade | Child stands on balance board. While following the avatar that appears on the screen and taking steps to the rhythm of the music, he tries to catch the shapes that appear on the screen with the remote control and nuc chuck at the same time. |
| 29 | Kinect Adventures | Kinect Adventures is an exergame for the Xbox 360 that uses the Kinect sensor to track player movements. Players engage in various adventure activities like rafting, obstacle courses, and more, by physically moving their bodies. |
| 30 | Kinect Sports | Kinect Sports is an exergame developed by Rare and published by Microsoft Studios for the Xbox 360. Utilizing the Kinect sensor, players engage in a variety of sports activities such as soccer, bowling, track and field, table tennis, boxing, and beach volleyball. |
| 31 | Just Dance | Just Dance is a rhythm and dance game developed by Ubisoft for the Nintendo Wii. Players follow on-screen dance moves to popular songs, using the Wii Remote to track their movements. |
| 32 | Mario and Sonic at the Olympic Games | Mario and Sonic at the Olympic Games is a sports video game developed by Sega for the Nintendo Wii. Featuring characters from the Mario and Sonic franchises, players compete in various Olympic events such as track and field, swimming, gymnastics, and more. |
| 33 | BKOOL Interactive Cycling | BKOOL Interactive Cycling is a virtual cycling simulation game that connects to the BKOOL cycling trainer and a tablet, such as the HUAWEI MediaPad T5. Players experience realistic cycling routes and environments, with the game adjusting resistance based on the terrain. |
| 34 | MobileKids Monster Manor | The MKMM game uses a monster character theme for its graphics and offers gold as a within-game prize to players to encourage them to venture through the game’s ascending levels. |
| 35 | Start the Party | Start the Party is a party game developed by Supermassive Games for the PlayStation 3. Utilizing the PlayStation Move controller, players engage in a variety of mini-games that involve motion-based interactions. |
| 36 | Eye Pet | EyePet is a virtual pet simulation game developed by London Studio for the PlayStation 3. Using the PlayStation Move and Eye input devices, players interact with their virtual pet by performing various activities like feeding, grooming, and playing games. |
| 37 | Sports Champions | Sports Champions is a sports simulation game developed by San Diego Studio for the PlayStation 3. It uses the PlayStation Move controller to allow players to participate in a variety of sports activities such as archery, table tennis, beach volleyball, and more. |
| 38 | Xbox Sport Season Series 1 and 2 | Kinect Sports: Season 1 and Kinect Sports: Season 2 are sports simulation games developed by Rare and published by Microsoft Studios for the Xbox 360. Utilizing the Kinect sensor, players can engage in various sports activities using full-body motion control. Season 1 includes sports such as soccer, bowling, track and field, table tennis, boxing, and beach volleyball. Season 2 adds new sports like tennis, golf, American football, baseball, skiing, and darts. |
| 39 | Beat Saber | Beat Saber is a virtual reality rhythm game developed by Beat Games. Players use VR motion controllers to slash through blocks representing musical beats with dual lightsabers. The game is known for its energetic gameplay, immersive music tracks, and visually stunning environments, providing a fun and engaging way to exercise and enjoy music. It is compatible with various VR systems, including the HTC Vive, Oculus Rift, and PlayStation VR. |
| 40 | Zumba | Zumba is a dance fitness program that combines Latin and international music with dance moves. Created by Colombian dancer and choreographer Alberto "Beto"Perez, Zumba routines incorporate interval training, alternating fast and slow rhythms, to improve cardiovascular fitness. |
| 41 | Wii Fit U minigames | Wii Fit U, developed by Nintendo for the Wii U console, includes a variety of minigames designed to make fitness fun and engaging. These minigames cover a range of activities, including balance games, strength training, aerobics, and yoga. |
| 42 | Your Shape Fitness Evolved 2012 | Your Shape: Fitness Evolved 2012 is a fitness game developed by Ubisoft for the Xbox 360. Utilizing the Kinect sensor, it provides a personalized workout experience with various fitness routines, including cardio, strength training, and flexibility exercises. |
| 43 | Interactive cycling | While cycling, the participants see a route on the screen. They can pick a route, and it mimics the experience of cycling outside, thus offering simultaneous physical and cognitive stimulation. |
| 44 | Wii Sports | Wii Sports is a sports simulation game developed by Nintendo for the Nintendo Wii console. Released in 2006, it includes five sports: tennis, baseball, bowling, golf, and boxing. Utilizing the Wii Remote's motion-sensing capabilities, players perform real-life movements to control their in-game actions, making the gameplay intuitive and engaging. |
| 45 | Wii Fit | Wii Fit is a fitness game developed by Nintendo for the Nintendo Wii console. Released in 2007, it includes a variety of exercises such as yoga, strength training, aerobics, and balance games. The game uses the Wii Balance Board, a platform peripheral that measures weight and balance, to provide an interactive and personalized fitness experience. |
| 46 | Shape up Season 2 | Shape Up: Season 2 is a fitness game developed by Ubisoft for the Xbox One. Utilizing the Kinect sensor, it offers a variety of fun and engaging workout routines that combine gaming elements with physical exercise. Players can participate in activities such as dance, strength training, and cardio challenges, all designed to make workouts more enjoyable and motivating. |
| 47 | Wii tennis | Wii Tennis is one of the sports included in the Wii Sports game developed by Nintendo for the Nintendo Wii console. In this game, players use the Wii Remote to simulate tennis strokes, swinging the controller as they would a real tennis racket. |
| 48 | Wii boxing | Wii Boxing is one of the sports included in the Wii Sports game developed by Nintendo for the Nintendo Wii console. Players use the Wii Remote and Nunchuk to simulate boxing movements, with each controller representing one of the player's fists. The game tracks punches, dodges, and blocks, providing an immersive and physical boxing experience. |
| 49 | Zombies, Run! | an immersive running game and audio adventure that instructs players to collect supplies and avoid being attacked by Zombies as they exercise. |
| 50 | The Walk | The Walk is also an audio adventure game that presents episodes and challenges to the player, who is tasked with a package that must be delivered in order to save the world. In order to stay alive, the player must walk/run the length of the United Kingdom. |
