# Supplementary material for: Exergame-Based Behavior Change Interventions for Promoting Physical Activity: Systematic Review and Meta-Analysis of Randomized Controlled Studies
Source: J Med Internet Res. 2025 Aug 8;27:e62906. doi: 10.2196/62906 (PMC12334110; doi:10.2196/62906)
Supplement: Multimedia Appendix 5 [file jmir-v27-e62906-s005.docx]

| BCT identified in each study | | | | | | | | | | |
| --- | --- | --- | --- | --- | --- | --- | --- | --- | --- | --- |
| **BCT taxonomy** | **Adamo**  **2010** | **Baranowski**  **2012** | **Campelo**  **2023** | **Cavusoglu**  **2023** | **Comeras-Chueca_**  **2022** | **Garde**  **2016** | **Howie**  **2015** | **Lau**  **2016** | **Sousa**  **2022** | **Swartz**  **2022** |
| 1.1 Goal setting (behaviour) |  |  |  | **√** | **√** |  | **√** | **√** |  | **√** |
| 1.2 Problem solving |  |  |  |  |  |  | **√** | **√** |  | **√** |
| 1.4 Action planning | **√** |  |  | **√** | **√** |  | **√** | **√** | **√** | **√** |
| 1.5 Review behaviour goal(s) |  |  |  |  |  |  |  |  |  |  |
| 2.1 Monitoring of behaviour by others without feedback |  | **√** | **√** | **√** |  |  |  | **√** |  |  |
| 2.2 Feedback on behaviour | **√** |  | **√** |  |  | **√** | **√** | **√** |  | **√** |
| 2.3 Self-monitoring of behaviour |  |  | **√** |  |  | **√** | **√** |  | **√** | **√** |
| 2.5 Monitoring of outcome(s) of behaviour without feedback | **√** |  |  |  |  |  |  |  |  |  |
| 3.1 Social support (unspecified) |  |  | **√** | **√** |  |  |  | **√** |  | **√** |
| 3.2 Social support (practical) |  |  |  |  |  | **√** | **√** | **√** |  |  |
| 3.3 Social support (emotional) |  |  |  |  |  | **√** |  |  |  |  |
| 4.1 Instruction on how to perform the behaviour |  |  | **√** |  |  |  |  | **√** | **√** | **√** |
| 5.1 Information about health consequences |  |  |  |  |  |  |  |  |  | **√** |
| 5.6 Information about emotional consequences |  |  |  |  |  |  |  |  |  | **√** |
| 6.1 Demonstration of the behaviour | **√** |  | **√** |  |  |  |  | **√** |  | **√** |
| 6.2 Social comparison |  |  |  |  |  | **√** |  |  |  | **√** |
| 7.1 Prompts/cues |  |  |  |  |  |  |  |  | **√** |  |
| 8.1 Behavioural practice/rehearsal |  |  |  |  |  |  |  |  |  | **√** |
| 8.7 Graded tasks |  |  | **√** | **√** | **√** | **√** |  |  | **√** | **√** |
| 9.1 Credible source |  |  | **√** |  |  |  |  |  |  | **√** |
| 10.2 Material reward (behavior) |  |  |  |  |  |  | **√** |  | **√** |  |
| 10.3 Non-specific reward |  |  |  |  |  | **√** |  | **√** |  | **√** |
| 10.4 Social reward |  |  |  |  |  | **√** |  |  |  |  |
| 11.3 Conserving mental resources |  |  | **√** |  |  |  |  |  | **√** |  |
| 12.1 Restructuring the physical environment |  |  |  |  |  |  |  | **√** |  |  |
| 12.2 Restructuring the social environment |  |  |  |  |  |  |  |  |  |  |
| 12.5 Adding objects to the environment |  | **√** | **√** | **√** | **√** |  | **√** | **√** |  |  |
| 14.4 Reward approximation |  |  |  |  |  |  |  |  |  |  |
| 14.5 Rewarding completion |  |  |  |  |  | **√** |  |  |  |  |
| **Total No. of BCT** | **4** | **2** | **10** | **6** | **4** | **9** | **8** | **12** | **7** | **15** |

| **Supplementary Table S6 BCT identified in each study (*continued*)** | | | | | | | | | | |
| --- | --- | --- | --- | --- | --- | --- | --- | --- | --- | --- |
| **BCT taxonomy** | **Trost**  **2014** | **van Santen**  **2020** | **Maloney**  **2008** | **Maloney**  **2012** | **Şimşek**  **2016** | **Bowling**  **2021** | **Kempf**  **2013** | **Lwin**  **2012** | **Cowdery**  **2015** | **Hamari**  **2019** |
| 1.1 Goal setting (behaviour) |  | **√** | **√** |  | **√** | **√** | **√** | **√** | **√** | **√** |
| 1.2 Problem solving |  |  | **√** | **√** |  | **√** |  |  |  |  |
| 1.4 Action planning |  | **√** | **√** | **√** | **√** | **√** | **√** | **√** |  | **√** |
| 1.5 Review behaviour goal(s) |  |  |  |  |  | **√** |  |  |  |  |
| 2.1 Monitoring of behaviour by others without feedback |  |  |  |  |  |  |  |  |  |  |
| 2.2 Feedback on behaviour |  | **√** |  |  | **√** | **√** |  |  |  |  |
| 2.3 Self-monitoring of behaviour |  |  | **√** | **√** | **√** | **√** |  |  | **√** | **√** |
| 2.5 Monitoring of outcome(s) of behaviour without feedback |  |  |  |  |  |  |  |  |  |  |
| 3.1 Social support (unspecified) |  |  | **√** |  |  | **√** |  |  | **√** | **√** |
| 3.2 Social support (practical) |  |  | **√** |  | **√** | **√** |  |  |  |  |
| 4.1 Instruction on how to perform the behaviour |  |  | **√** | **√** | **√** | **√** | **√** |  | **√** | **√** |
| 5.1 Information about health consequences |  |  |  |  |  |  |  |  |  |  |
| 5.6 Information about emotional consequences |  |  |  |  |  |  |  |  |  |  |
| 6.1 Demonstration of the behaviour |  |  |  |  |  |  |  |  |  |  |
| 6.2 Social comparison |  |  | **√** |  |  |  |  |  |  |  |
| 7.1 Prompts/cues |  |  |  |  |  |  |  |  |  |  |
| 8.1 Behavioural practice/rehearsal |  |  |  |  |  |  |  |  |  |  |
| 8.7 Graded tasks |  |  |  |  |  | **√** |  | **√** |  |  |
| 9.1 Credible source |  |  | **√** |  |  |  |  |  |  |  |
| 10.2 Material reward (behavior) |  |  |  |  |  |  |  |  |  |  |
| 10.3 Non-specific reward |  |  |  |  |  |  |  |  |  |  |
| 10.4 Social reward |  |  |  |  |  | **√** |  |  |  |  |
| 11.3 Conserving mental resources |  | **√** |  |  |  |  |  |  |  |  |
| 12.1 Restructuring the physical environment |  | **√** |  |  | **√** |  |  |  |  |  |
| 12.2 Restructuring the social environment |  | **√** |  |  |  |  |  |  |  |  |
| 12.5 Adding objects to the environment | **√** | **√** | **√** | **√** |  | **√** | **√** |  | **√** |  |
| 14.4 Reward approximation |  |  |  |  |  | **√** |  |  |  |  |
| 14.5 Rewarding completion |  |  |  |  |  |  |  |  |  |  |
| **Total No. of BCT** | **1** | **7** | **10** | **5** | **7** | **13** | **4** | **3** | **5** | **5** |
