# Supplementary material for: Exergame-Based Behavior Change Interventions for Promoting Physical Activity: Systematic Review and Meta-Analysis of Randomized Controlled Studies
Source: J Med Internet Res. 2025 Aug 8;27:e62906. doi: 10.2196/62906 (PMC12334110; doi:10.2196/62906)
Supplement: Multimedia Appendix 6 [file jmir-v27-e62906-s006.pptx]

## Slide 1
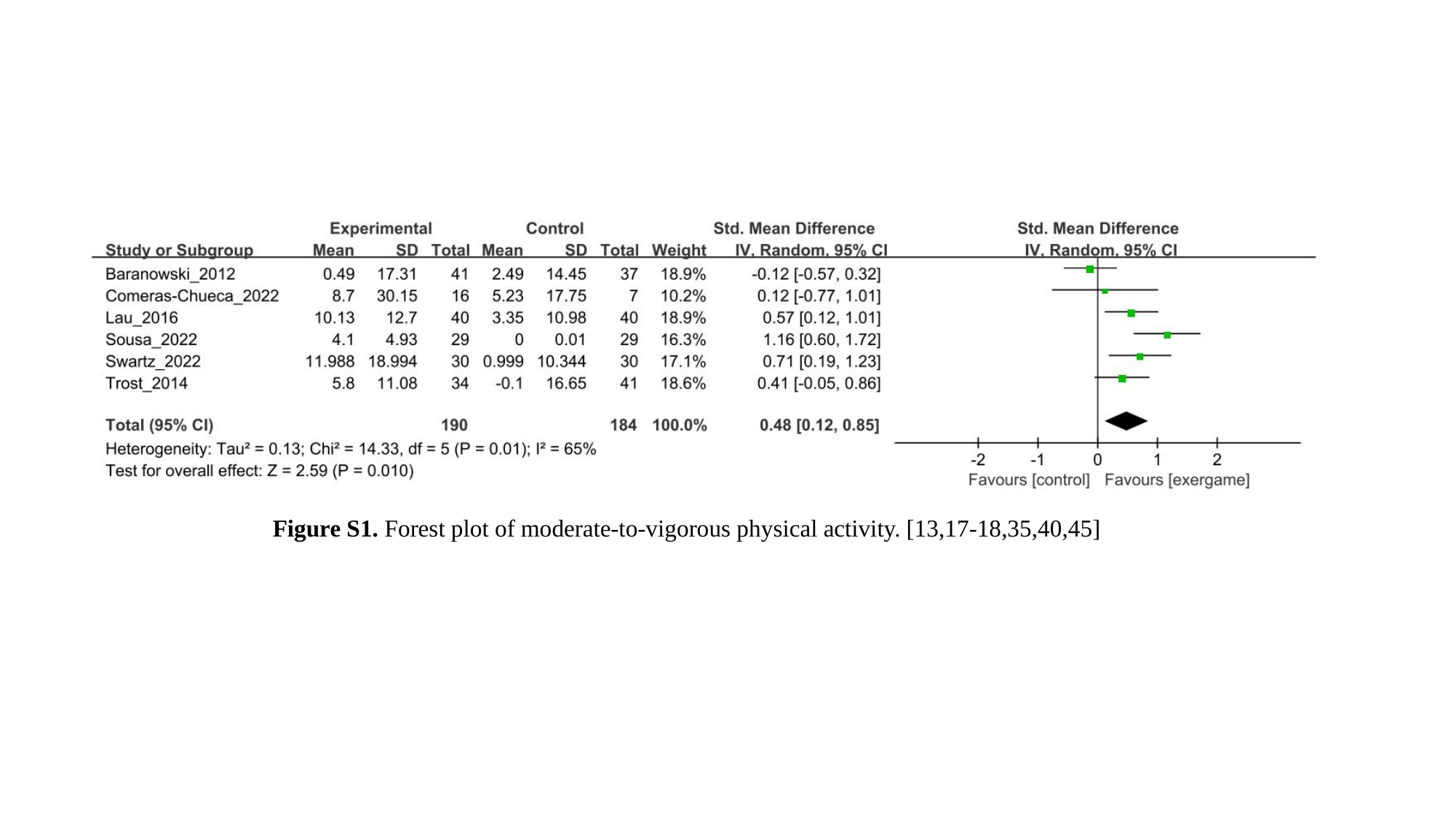

Figure S1. Forest plot of moderate-to-vigorous physical activity. [13,17-18,35,40,45]

## Slide 2
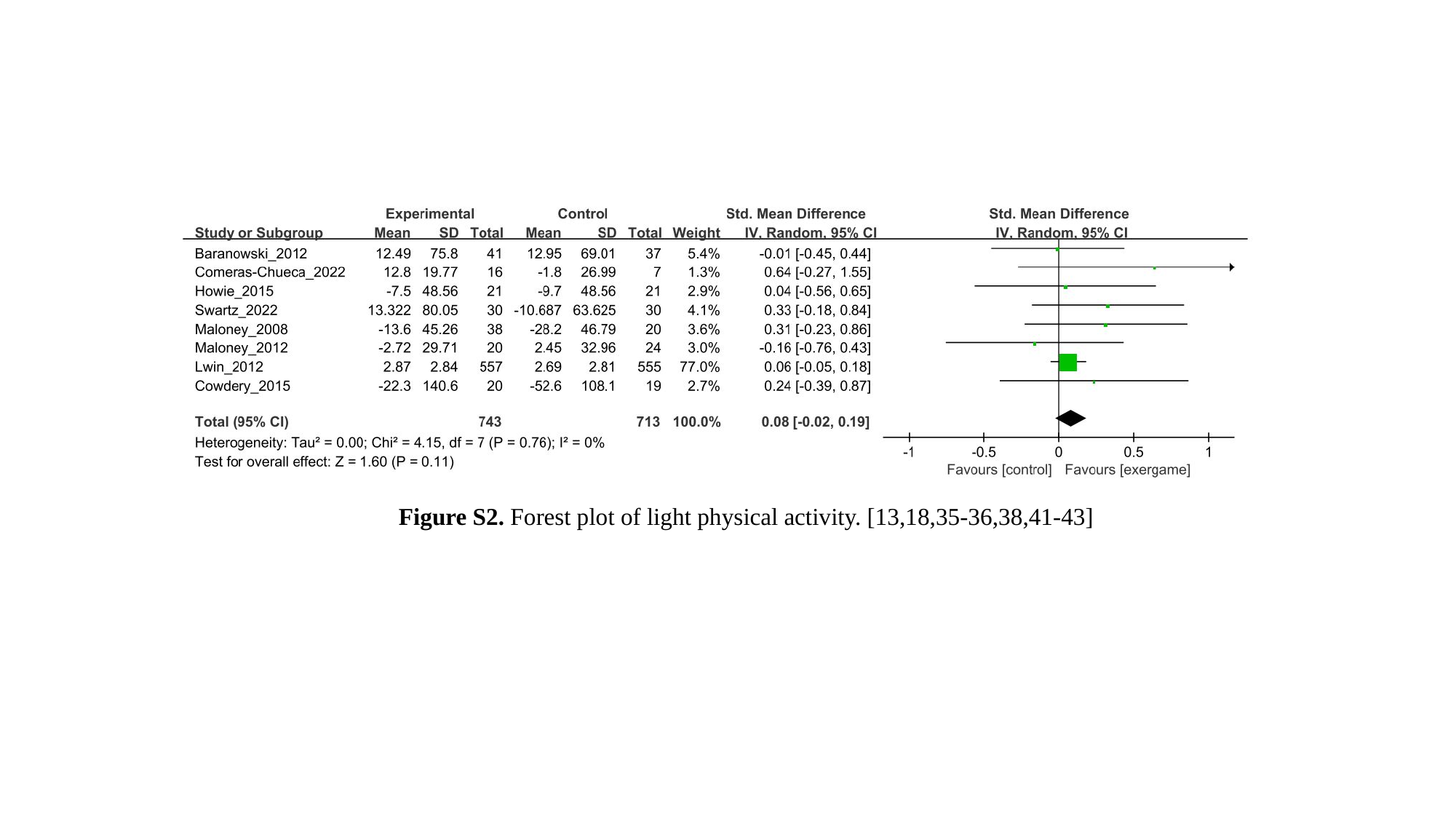

Figure S2. Forest plot of light physical activity. [13,18,35-36,38,41-43]

## Slide 3
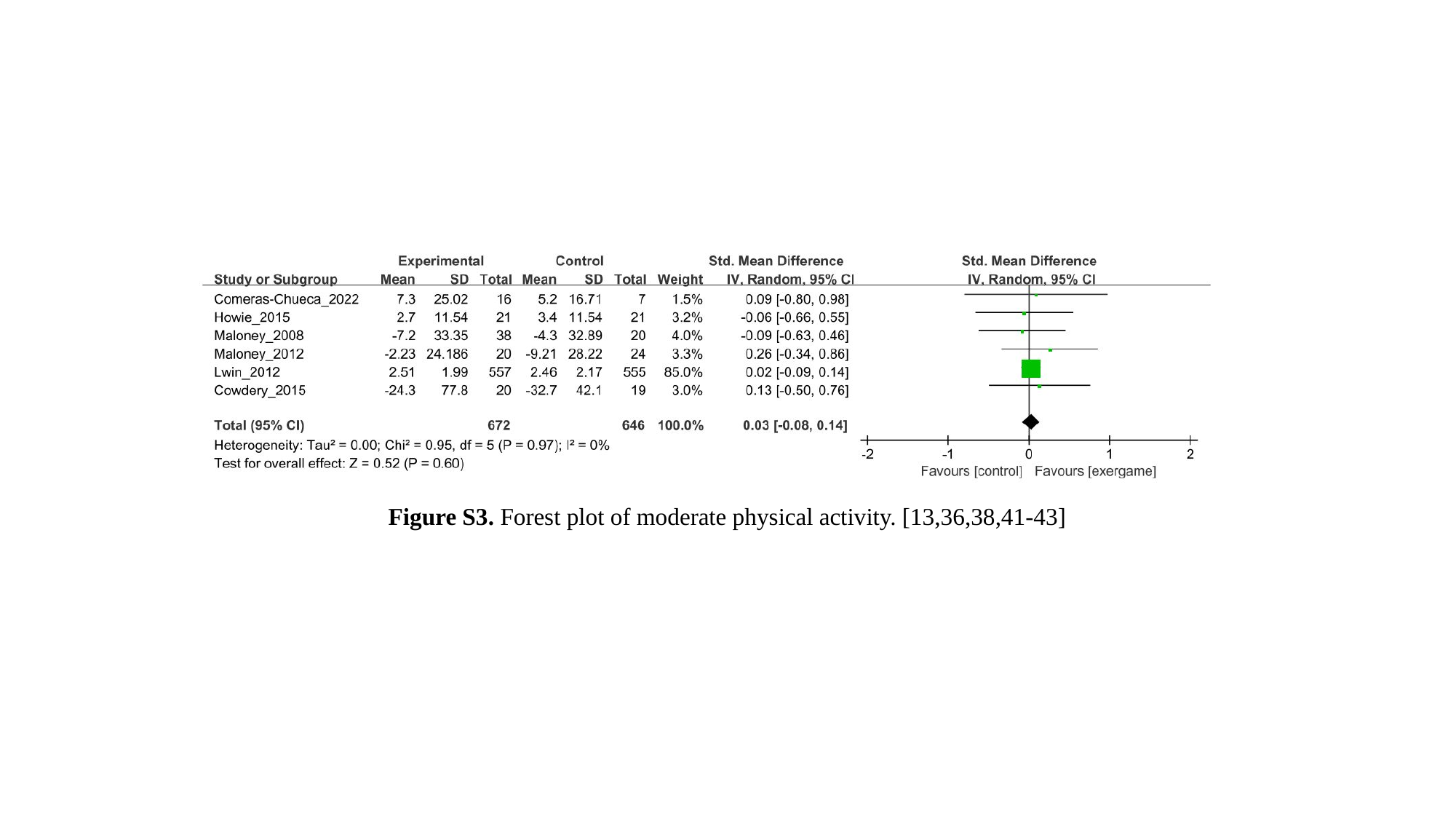

Figure S3. Forest plot of moderate physical activity. [13,36,38,41-43]

## Slide 4
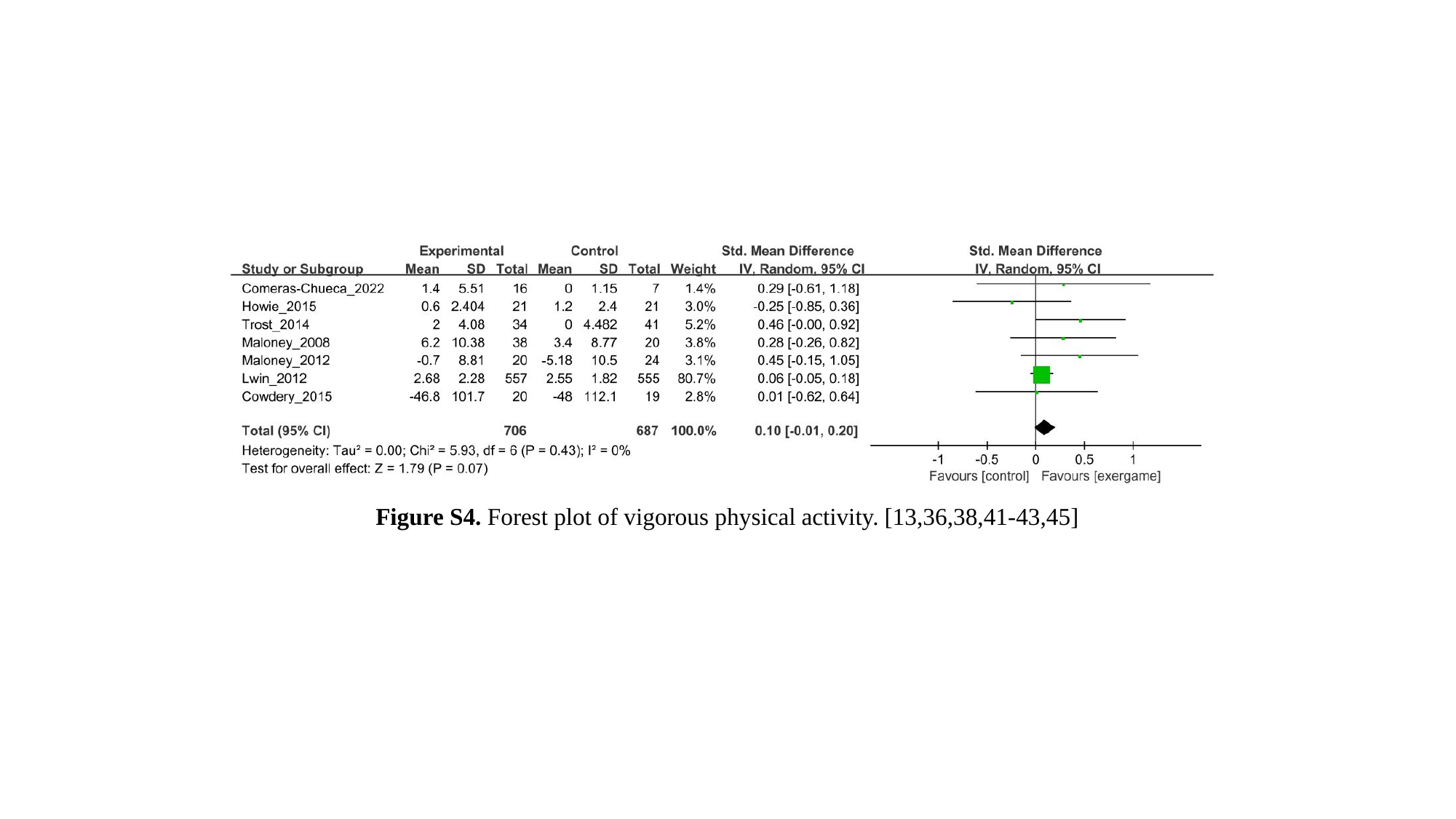

Figure S4. Forest plot of vigorous physical activity. [13,36,38,41-43,45]

## Slide 5
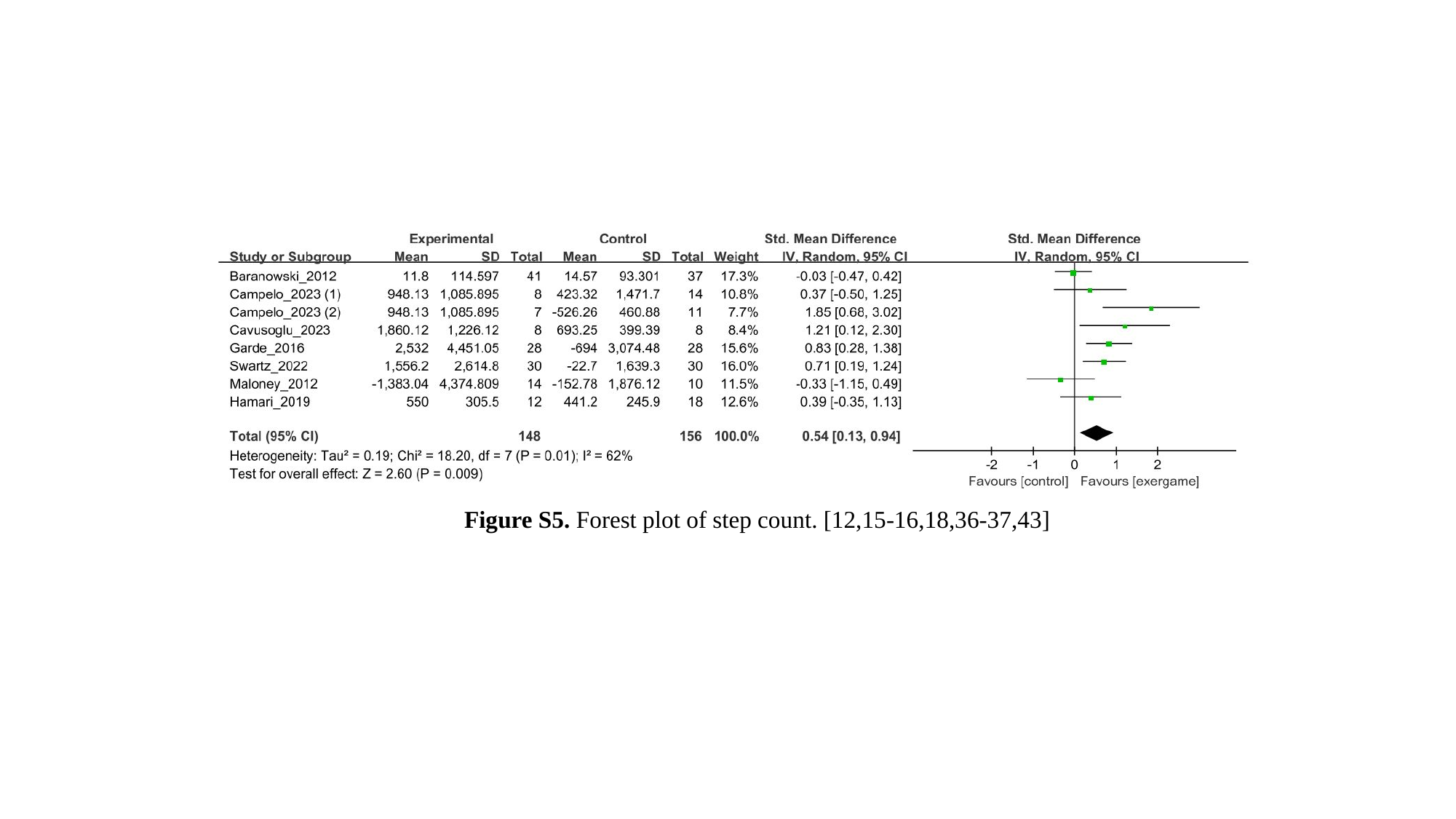

Figure S5. Forest plot of step count. [12,15-16,18,36-37,43]

## Slide 6
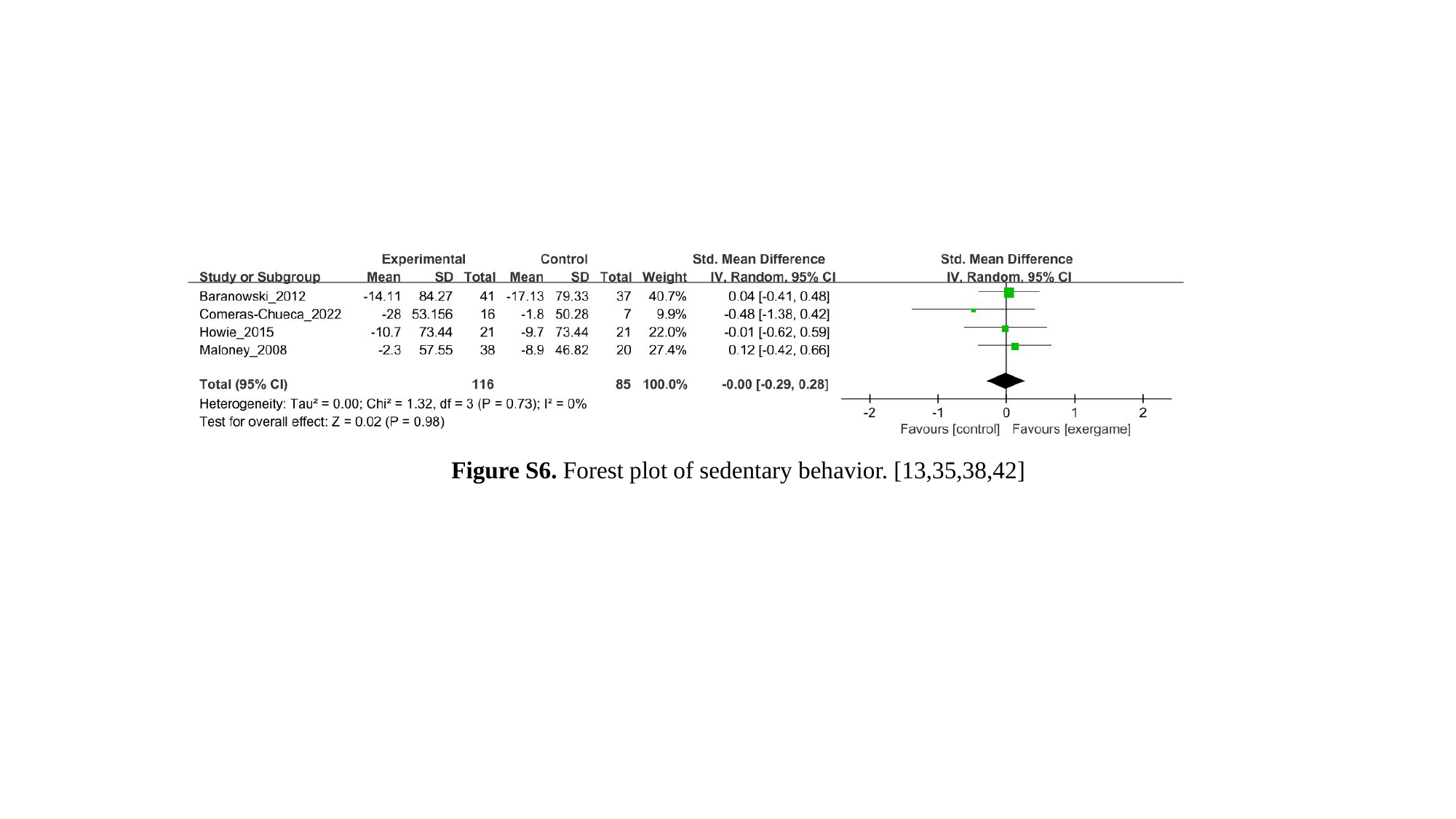

Figure S6. Forest plot of sedentary behavior. [13,35,38,42]

## Slide 7
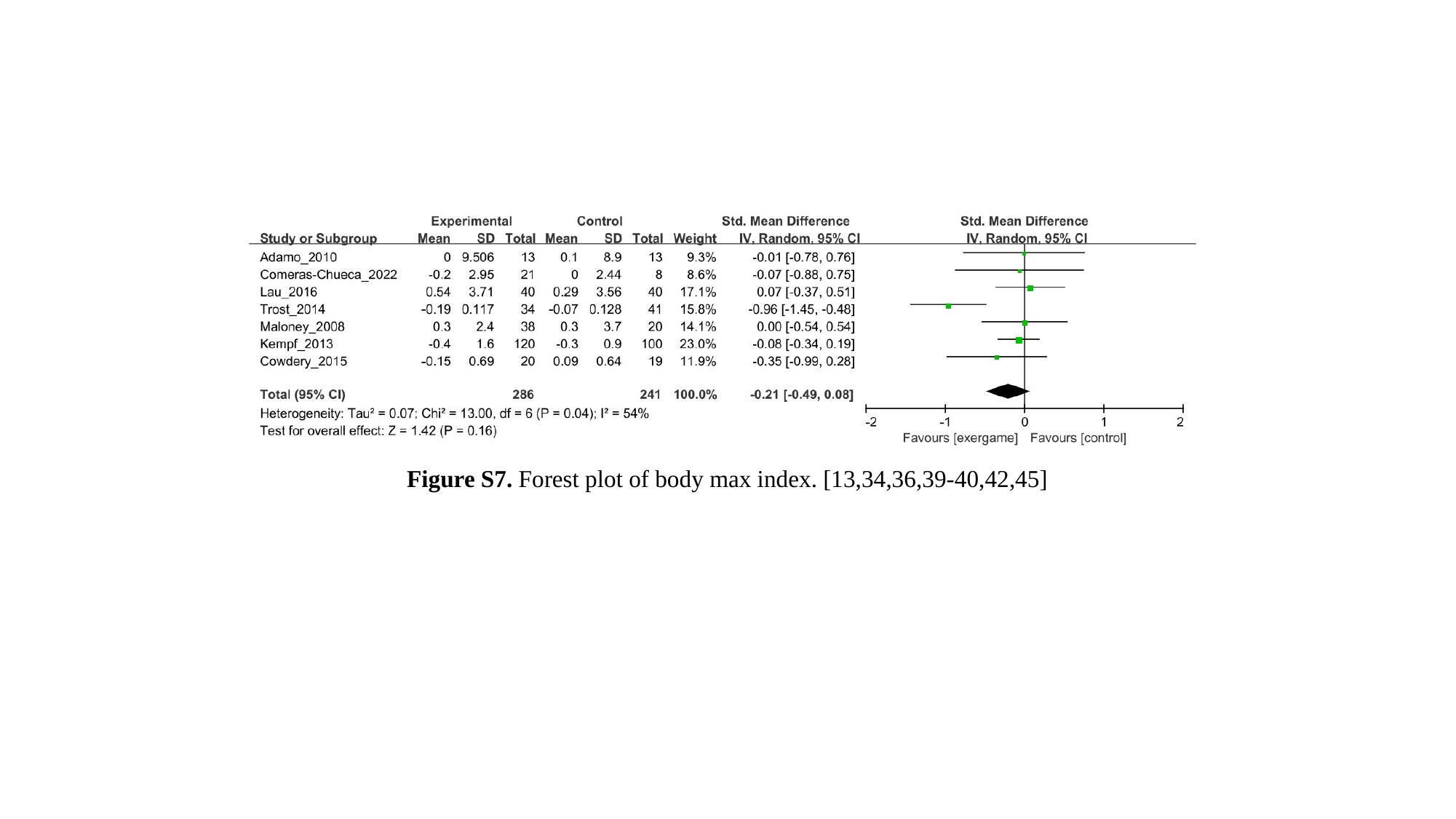

Figure S7. Forest plot of body max index. [13,34,36,39-40,42,45]
